# IL-17A deficiency in HLA-DR3 transgenic mice enriches beneficial *Prevotella* species in gut to promote Tregs and reduce CNS autoimmunity

**DOI:** 10.1186/s40168-026-02394-w

**Published:** 2026-06-29

**Authors:** Shailesh K. Shahi, Sudeep Ghimire, Samantha N. Jensen, Peter C. Lehman, Allison G. Rux, Souradip Sinha, Nicholas Borcherding, Munir R. Tanas, Katherine N. Gibson-Corley, Sukirth M. Ganesan, Nitin J. Karandikar, Ashutosh K. Mangalam

**Affiliations:** 1 Department of Pathology, Carver College of Medicine, University of Iowa, Iowa City, USA; 2 Graduate Program in Immunology, University of Iowa, Iowa City, USA; 3 Molecular Medicine Graduate Program, University of Iowa, Iowa City, USA; 4 Center for Biocatalysis and Bioprocessing, University of Iowa, Iowa City, USA; 5 Department of Pathology and Immunology, Washington University School of Medicine, Saint Louis, MO USA; 6 Present Address: Department of Pathology, Microbiology and Immunology, Vanderbilt University Medical Center, Nashville, TN USA; 7 Department of Periodontics, College of Dentistry, University of Iowa, Iowa City, USA; 8 Iowa City VA Health Care System, Iowa City, IA 52246 USA; 9 Present Address: Present address- Department of Microbiology and Immunology, Weill Cornell Medicine, Cornell University, New York, USA

**Keywords:** Immune regulation, IL-17A, Gut microbiota, *Prevotella*, Tregs, Experimental Autoimmune Encephalomyelitis (EAE), Multiple sclerosis

## Abstract

**Background:**

Human Leukocyte Antigen (HLA) class-II genes, particularly HLA-DR2 and HLA-DR3, and the gut microbiota are intricately linked to the pathobiology of multiple sclerosis (MS) through their ability to regulate host immunity, a critical factor in disease pathogenesis. An imbalance between anti-inflammatory CD4^+^ Tregs and pro-inflammatory IL-17A-secreting CD4^+^ Th17 cells is thought to drive disease. However, a key unresolved question is whether HLA-class II-restricted CD4^+^IL-17A cells can influence Treg populations and the extent to which gut microbiota regulate this IL-17A-Treg axis. Therefore, we utilized humanized transgenic mice expressing the HLA class-II gene and deficient in mouse class-II molecules, where all CD4^+^ T cells are selected on the human HLA class-II molecule, closely mimicking human immune responses.

**Results:**

Utilizing IL-17A-deficient (DR3.IL-17A^−/−^) mice expressing HLA-DR3 (HLA-DRβ1*0301), we show that IL-17A deficiency enriches beneficial gut bacteria, including *Prevotella species*, enhances peroxisome proliferator–activated receptor (PPAR) signaling, and increases FoxP3^+^ regulatory T (Treg) cells and IL-10 production. The importance of gut microbiota in promoting Tregs and anti-inflammatory responses was confirmed by administering *Prevotella copri*, a common commensal in human gut, which mirrored the effects observed in IL-17A-deficient mice by inducing PPAR signaling and Treg population. Moreover, DR3.IL-17A^−/−^ mice exhibited a marked reduction in EAE severity compared to IL-17A-sufficient (DR3) mice, underscoring the enhanced functional capacity of the Treg population in mitigating disease progression. Cohousing experiments validated the role of gut microbiota in immune regulation including Treg induction, as demonstrated by the transfer of *Prevotella species* from IL-17A-deficient mice to IL-17A-sufficient mice, increased Treg populations and attenuated EAE severity in recipient DR3 mice.

**Conclusions:**

This study redefines IL-17A's role in immune regulation, emphasizing its ability to directly influence gut microbiota composition and the abundance of Treg-promoting bacteria. Thus, gut microbiota-targeted therapies, particularly those promoting Treg-inducing bacteria like *Prevotella species*, hold promise for treating autoimmune diseases by modulating host immune responses.

Video Abstract

**Supplementary Information:**

The online version contains supplementary material available at 10.1186/s40168-026-02394-w.

## Main text

Multiple sclerosis (MS), a chronic, inflammatory, neurodegenerative disease of the central nervous system (CNS), is driven by both genetic and environmental factor. Among the genetic factors, HLA class-II genes, particularly HLA-DR2 and HLA-DR3, show the strongest association with MS [1–5]. HLA class-II restricted CD4^+^ T helper (Th) cells of the Th1 and/or Th17 phenotype play an important role in pathobiology of the disease. IL-17A a key pro-inflammatory cytokine of Th17 subset, is essential for resolving bacterial and fungal infections [6] but can lead to unresolved inflammation and tissue damage in autoimmune diseases like MS [7–12]. Despite its established role, the precise mechanisms by which IL-17A influences MS pathogenesis including their ability regulate Tregs remain poorly understood.

The relatively low concordance rates in monozygotic (30%) and even lower rates in dizygotic twins suggest a significant contribution (up to two-thirds) from environmental factors. While several environmental factors have been implicated, including EBV, the gut microbiome has emerged as a key factor due to its ability to regulate host immune responses. The microbiome maintains immune homeostasis by modulating the balance between CD4^+^CD25^+^FoxP3^+^ regulatory T cells (Tregs) and pro-inflammatory Th17 cells [13, 14]. Germ-free mouse studies have shown that gut bacteria are essential for restoring both Tregs and Th17 cells, highlighting their role in immune regulation [15, 16]. Adminstering single bacterium such as segmented filamentous bacterium have shown to restore Th17 [17] population and a mixture of chloroform-resistant *Clostridium* can restore Treg population [18]. Additionally mucosal biospies from people with MS have been shown to have inverse correlation between relative abudance of *Prevotella* and Th17 cells. These studies highlight the important role of gut microbiota in the regulation of the Th17 and Treg population and their role in the pathobiology of MS/EAE. The interplay between Th17, Treg, and gut microbiota is poorly understood as prior study have shown that the effect of IL-17 can modulate gut microbiota and alter CNS autoimmunity [19] independent of Treg modulation in wild type C57BL/6 (B6) mice.

To better understand the interplay between genetic factors (HLA class II), environmental factors (gut microbiome), and host immune responses in the pathobiology of MS, an animal model incorporating human HLA class-II is crucial. We previously generated HLA class-II transgenic mice that lack endogenous mouse MHC class-II molecules, resulting in a CD4^+^ T cell repertoire exclusively restricted to human HLA class-II molecules, thereby closely mimicking the human immune response and validating the significance of HLA class-II molecules in the pathogenesis of MS [20–24]. Our previous work demonstrated that HLA class II polymorphisms influence disease incidence and susceptibility, and shape gut microbiota composition, highlighting the importance of this model system [25–29]. To specifically investigate the IL-17A-Treg-microbiome axis, particularly IL-17A's ability to regulate Treg populations through the gut microbiota, we generated IL-17A-deficient HLA-DR3 transgenic mice (DR3.IL-17A^−/−^).

We show that IL-17A deficiency was associated with an enrichment of *Prevotella* and related bacteria and activation of the Treg-promoting PPAR signaling pathway in the gut. DR3.IL-17A^−/−^ mice exhibited increased CD4^+^CD25^+^FoxP3^+^ Tregs and IL-10 production, suggesting IL-17A’s role in modulating Tregs through microbiota. Administration of *Prevotella copri* to DR3 mice replicated these effects by activating PPAR signaling and enhancing Treg levels. Additionally, cohousing DR3 mice with DR3.IL-17A^−/−^ mice led to the transfer of *Prevotella species*, increased Tregs, and reduced EAE severity in recipient mice. Collectively, our findings demonstrate that IL-17A regulates Treg abundance and immune homeostasis by modulating *Prevotella* and other simialr gut bacteria. This IL-17A–*Prevotella*–Treg axis provides mechanistic insights into EAE pathogenesis and highlights the potential for microbiota-targeted therapies in managing MS.

## Results

### IL-17A deficiency modulates the gut microbiome to enrich *Prevotella* and related species

We generate HLA-DR3.IL-17A^−/−^ deficient transgenic mice (referred as DR3.IL-17A^−/−^ here on) and maintained at University of Iowa mouse facility as described in methods. All transgenic mice developed normally with no obvious signs of pathology, and the analysis of splenocytes confirmed that these DR3.IL-17A^−/−^ mice lacked IL-17A-expressing CD4^+^ T cells (Fig. S1).

**Fig. 1:**
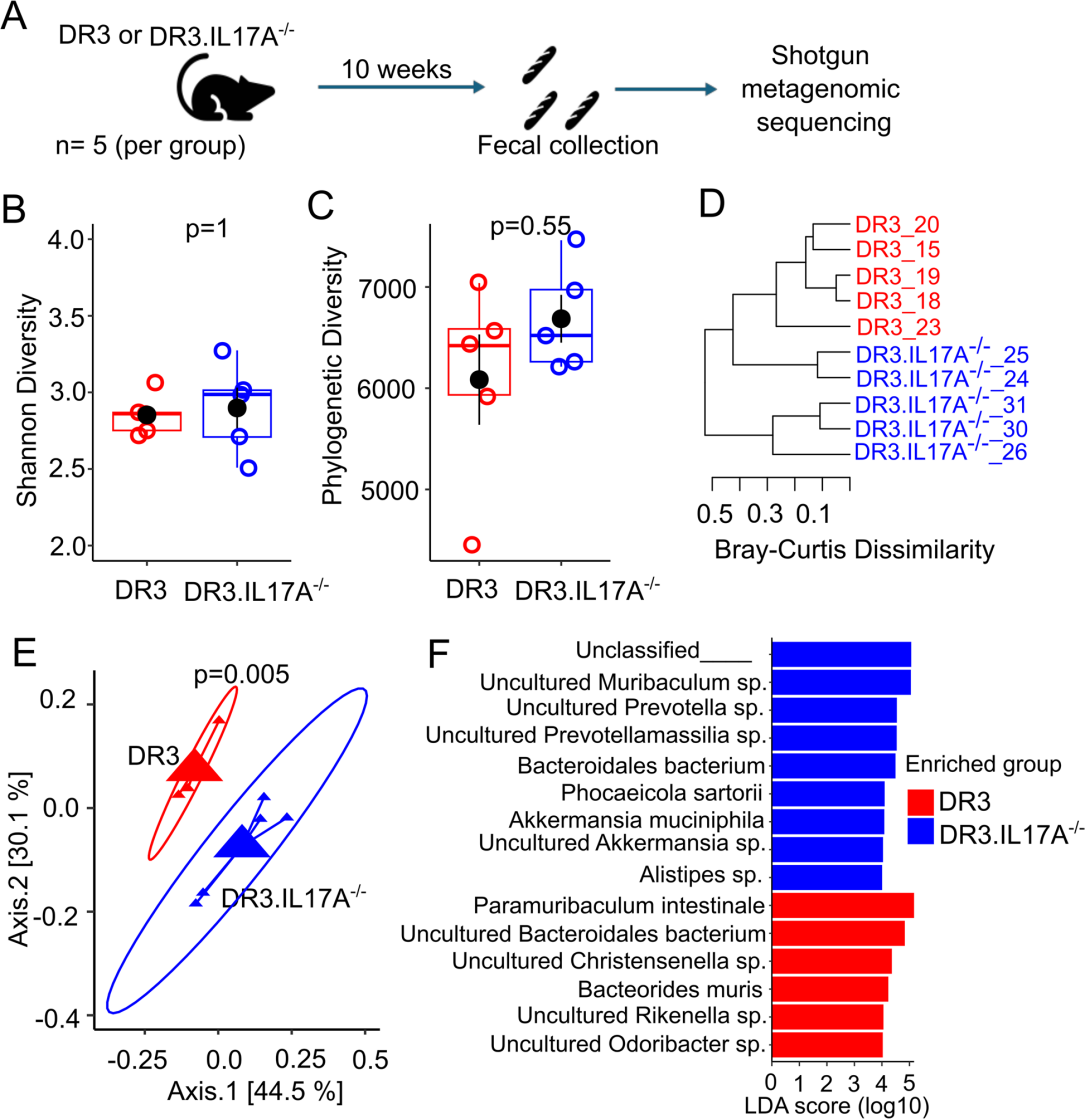
DR3 and DR3.IL-17A^−/−^ transgenic mice harbor distinct gut microbiome. Fecal samples were collected from 10-week-old DR3 (n = 5) and DR3.IL-17A^−/−^ (n = 5) female mice. Gut microbiota composition was analyzed using shotgun metagenomic sequencing of fecal DNA. **A** Schematic of experimental design representing fecal collection and gut microbiota analysis. **B** Shannon and **C** Phylogenetic diversity in DR3 and DR3.IL-17A^−/−^ mice. **D** Bray–Curtis dissimilarity based dendrogram showing distinct separation of DR3 and DR3.IL-17A^−/−^ gut microbiome using *hclust* clustering and ward.D2 method. **E** Beta-diversity PCoA plot depicting clustering of DR3 and DR3.IL-17A^−/−^ gut microbiome using Bray–Curtis dissimilarity metric. **F** Differential abundance of bacteria at species level using *lefse* from *microbiomeMarker* package at *kw_cutoff* = 0.01, *wilcoxon_cutoff* = 0.01 and *lda_cutoff* = 4 in R. Wilcoxon-test was performed for B, C and *adonis2* test was performed for E

The role of IL-17 in shaping gut microbiome composition and subsequently modulating the immune response to influence EAE susceptibility has been described previously [19, 30]. Although, specific bacterial altered were identified [19], the study was constrained by amplicon-based sequencing, and the exact role of IL-17A remained unclear. Thus, to overcome these shortcomings, we maintained DR3 and DR3.IL-17A^−/−^ mice on normal chow diet for 10 weeks, collected their fecal samples, and performed shotgun metagenomic sequencing on the fecal DNA (Fig. 1A). Fecal samples from the DR3 and DR3.IL-17A^−/−^ mice showed no significant differences in the α-diversity as measured by Shannon and phylogenetic diversity (Fig. 1B, C). However, the gut microbiome composition of DR3 mice clustered significantly distinclty from DR3.IL-17A^−/−^ mice (Fig. 1D, E). Notably, we found sixteen bacterial species; mostly uncultured, that were differentially abundant between the DR3 and DR3.IL-17A^−/−^ mice (Fig. 1F). Specifically, uncultured *Prevotella*-related species such as, *Prevotella* and *Prevotellamassilia* species and *Akkermansia* species were enriched in DR3.IL-17A^−/−^ mice while *Paramuribaculum intestinale*, uncultured *Bacteroides* bacterium, and uncultured *Christensenella* species were overrepresented in DR3 mice. Functionally, the gut microbiome composition of DR3 mice was significantly distinct from DR3.IL-17A^−/−^ mice (Fig. S2). LEfSe analysis showed differential enrichment of six metabolic pathways between the two groups (Fig. S2). Especially, pathways such as PWY-6609: adenine and adenosine salvage II, NAGLIPASYN-PWY: lipid IVA biosynthesis and GLCMANNANAUT-PWY: superpathway of N-acetylglucosamine, N-acetylmannosamine and N-acetylneuraminate degradation along with the other UNMAPPED pathways were enriched in DR3 while PWY-3781: aerobic respiration I and other unintegrated pathways were enriched in DR3.IL-17A^−/−^ mice.

### IL-17A deficient and *Prevotella copri* treatment both enrich PPAR signaling in DR3 mice

**Fig. 2:**
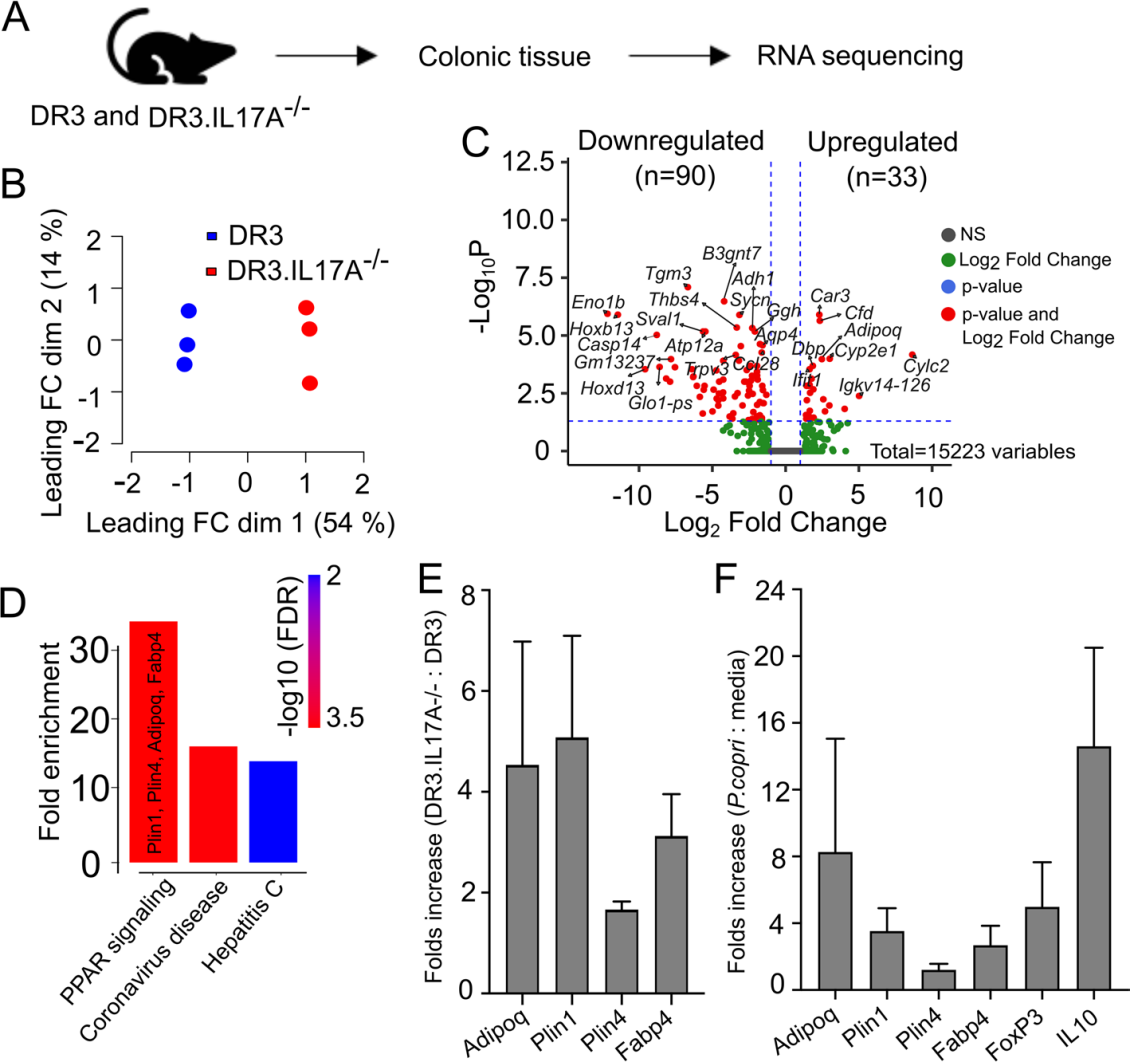
DR3 and DR3.IL-17A^−/−^ transgenic mice exhibit distinct anti-inflammatory gene expression patterns in the colon. Colonic tissue was harvested from 10-week-old DR3 (n = 3) or DR3.IL-17A^−/−^ female mice (n = 3) and subjected to bulk RNA sequence analysis. **A** Schematic diagram of workflow. **B** Multi-dimensional scaling plot showing the separation of DR3 and DR3.IL-17A^−/−^ colonic samples. Each dot represents an RNA-Sequence of a sample. Sample groups are indicated by different colors, as indicated in the legend. **C** Volcano plot displaying the pattern of gene expression of DR3.IL-17A^−/−^ mice relative to DR3 mice. Genes that are significantly differentially expressed, with a Log_2_ fold change of > 1 and an FDR-corrected p-value < 0.05, are highlighted in red, blue lines represents the boundary for Log_2_ fold change and the adjusted p-value cutoff. **D** Heatmap of enriched genes in PPAR signaling pathway. **E** Ratio of average fold change expression of genes using qPCR related to PPAR signaling pathway in DR3.IL-17A^−/−^ to DR3 mice. **F** Effect of *Prevotella copri* (*P. copri*) DSM 18205 supplementation in the colon of DR3 mice**.** Mice (n = 5 in each group) were treated with either *P. copri* DSM 18205 (10^7^ CFU) or media (control) for every alternate day for two weeks. After two weeks, mice were euthanized, and RNA was isolated from the colonic tissue. Genes related to PPAR pathways along with FoxP3 and IL-10 were quantified using qPCR. Ratio of average fold change expression of genes related to PPAR signaling pathway along with FoxP3 and IL10 in *P. copri* treated to media treated mice. Bars represent the mean and standard error of the mean from each experimental group. *p*-value was determined by pairwise Mann–Whitney test

**Fig. 3:**
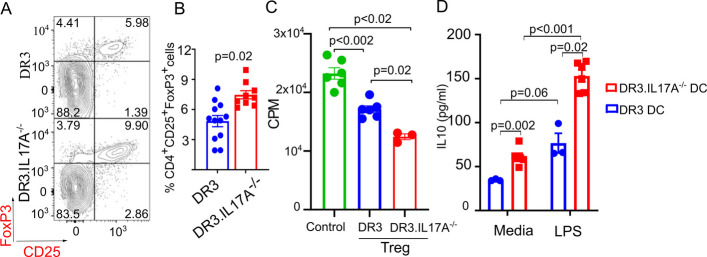
IL-17A deficiency in DR3 mice leads to the expansion of CD4^+^CD25^+^FoxP3^+^ regulatory T cells. Blood samples were collected from eight to ten-week-old DR3 and DR3.IL-17A^−/−^ mice via retro-orbital bleeding. Peripheral blood mononuclear cells (PBMCs) were isolated, stained with CD4, CD25, and FoxP3 antibodies and analysed by flow cytometry. **A** Representative flow cytometric plots of PBMCs from DR3 and DR3.IL-17A^−/−^ mice. Plots were gated on live lymphocytes and singlets. **B** Percentage of CD4^+^CD25^+^FoxP3^+^ Treg cells in PBMCs from mice shown in A. **C** Counts per minute (CPM) of DR3 mice CD4^+^ effector T cells and antigen presenting cells cultured with CD4^+^CD25^+^ Treg from the spleen of naïve DR3 or DR3.IL-17A^−/−^ mice. **D** Quantification of IL-10 cytokine production in naïve dendritic cells from DR3 and DR3.IL-17A^−/−^ mice following stimulation with LPS. Splenocytes were harvested from naïve 8–10-week-old HLA-DR3 (n = 3) and DR3.IL-17A.^−/−^ mice (n = 3) and CD11c + dendritic cells were isolated using BD™ IMag Particles. Purified CD11c + cells were activated with LPS for 24 h and IL-10 was measured by ELISA. Media without LPS was used as control. Bars represent the mean and standard error of the mean from two independent experiments. n = 4–8 mice per group for A-D. *p*-value was determined by unpaired t-test with Welch correction (B,C,D), and pairwise Mann–Whitney test for (D)

Next, we performed transcriptome analysis in the colonic tissues from DR3 and DR3.IL-17A^−/−^ mice (Fig. 2A). We observed distinct clustering between the DR3 and DR3.IL-17A^−/−^ mice (Fig. 2B). In the colon, 123 genes were differentially expressed between DR3 and DR3.IL-17A^−/−^ mice (Fig. 3C, Table S1). Thirty-three out of 123 differentially expressed genes were upregulated in DR3.IL-17A^−/−^ mice relative to DR3 mice. To gain insight into the biological role of the genes influenced by the absence of IL-17A, we looked into the KEGG pathways. Specifically, PPAR signaling pathway was > 30 folds enriched and consisted of *Adipoq, Plin1, Plin4,* and *Fabp4* genes (Fig. 2D). Using qPCR, *Plin1, Plin4* and *Fabp4* and *Adipoq* were validated to be enriched with an average 5.07, 5.39, 1.66, 3.12 folds in the colon of DR3.IL-17A^−/−^ compared to DR3 mice (Fig. 2E). To further confirm these transcriptomics and qPCR findings at protein level, we performed Western blot analysis on colonic lysates from wild-type DR3 and DR3.IL-17A^−/−^ mice. Immunoblotting with specific antibodies revealed elevated protein expression of Perilipin 1 (Plin1) and PPAR-γ in DR3.IL-17A^−/−^ mouse colons compared with DR3 controls (Fig. S3). These results provide direct evidence that loss of IL-17A enhances PPAR signaling in the colon, supporting our hypothesis that IL-17A deficiency promotes an anti-inflammatory, metabolically regulatory environment.

PPAR signaling regulate lipid/cholesterol metabolism and can also influence immune response [31]. While PPAR activation can promote Treg differentiation [32] and anti-inflammatory responses through modulation of Th1/Th17 response [33, 34], it has also been linked to T-cell survival and Th1/Th17 differentiation, highlighting its context-dependent functions [35, 36]. In DR3.IL-17A^−/−^ mice, we observed a selective upregulation of PPAR pathway genes, including key immunometabolic regulators, which coincided with reduced inflammatory signatures and enhanced Treg-associated pathways. These data suggest that, in the absence of IL-17A, PPAR signaling is skewed toward an immunoregulatory program rather than a Th1/Th17-effector phenotype, supporting a role for PPAR pathways in the anti-inflammatory immune environment observed in this model.

As uncultured *Prevotella* species were the most enriched bacterial taxa in DR3.IL-17A^−/−^ mice, we analyzed their potential contribution to the observed immunoregulatory phenotype. In addition to being one of the most prevalent *Prevotella* species in the human gut (~ 40%) [37], *P. copri* is specifically depleted in MS patients, as demonstrated in our recent human microbiome study [38]. In that study, *P. copri* abundance was significantly higher in HC than in MS patients, and oral administration of *P. copri* to microbiota-depleted mice ameliorated EAE severity [38]. Based on these findings, and because *Prevotella*-related taxa were enriched in DR3.IL-17A⁻/⁻ mice, we selected *P. copri* as a clinically relevant representative species to test whether its introduction into HLA-DR3 mice could recapitulate the immunoregulatory phenotype observed in IL-17A-deficient animals.

To test this, DR3 mice were gavaged with *P. copri* DSM 18205 (10^7^ CFU) or media control every other day for two weeks. Quantitative PCR analysis of colonic tissue showed significant upregulation of key PPAR pathway genes, including *Adipoq* (8.3-fold), *Plin1* (3.5-fold), *Fabp4* (1.2-fold), and *Plin4* (2.7-fold), compared to controls (Fig. 2F, Table S2). Moreover, *Foxp3* and *IL-10* transcripts were enriched 4.9- and 14.6-fold, respectively (Fig. 2F, Table S2), indicating a shift toward an anti-inflammatory immune environment. Consistent with these findings, our previous work showed that treatment with *Prevotella* species induced CD4⁺FoxP3⁺ Tregs in the colonic lamina propria and attenuated EAE severity in HLA class II transgenic mice [39]. Together, these data suggest that *P. copri* administration enhances PPAR signaling and promotes Treg and IL-10 expression.

### IL-17A deficiency enhances CD4^+^CD25^+^FoxP3^+^ Treg proliferation and dendritic cells (DCs)-derived IL-10

Previous study have demonstrated that CD4^+^CD25^+^FoxP3^+^ Treg cells can regulate autoimmune inflammation in the body and suppress IL-17A-secreting CD4^+^ Th17 cells [40, 41]. Our findings indicate that the increase abundance of *Prevotella* species in the gut microbiota, along with *P. copri* treatment induced FoxP3 and IL-10. These findings suggest the existence of a *Prevotella*–Treg-IL-10 axis that helps regulate the immune response in the absence of IL-17A.

Given that CD4^+^CD25^+^FoxP3^+^ Treg cells can suppress Th17-mediated EAE severity, we analyzed the frequency of CD4^+^CD25^+^FoxP3^+^ Treg cells in the peripheral blood of DR3 and DR3.IL-17A^−/−^ mice (Fig. 3A). Notably, DR3.IL-17A^−/−^ mice had a significantly higher CD4^+^CD25^+^Foxp3^+^ Treg cells compared to DR3 mice (Fig. 3A, B). In addition, CD4^+^CD25^+^ Treg cells isolated from the spleen of naïve DR3.IL-17A^−/−^ mice showed significantly higher suppressive ability against antigen specific CD4^+^CD25^−^ effector T cells than those derived from DR3 transgenic mice (Fig. 3C), further supporting the role of Treg in determining clinical EAE outcomes in DR3.IL-17A^−/−^ mice. Collectively, our data indicate that deficiency of IL-17A results in a higher frequency and number of CD4^+^CD25^+^ Treg cells with a higher suppressive ability.

In addition, to investigate the involvement of IL-10, we analyzed the production of IL-10 from LPS stimulated DCs isolated from naïve DR3 and DR3.IL-17A^−/−^ mice. Interestingly, CD11c^+^ DCs from naïve DR3.IL-17A^−/−^ mice produced significantly higher levels of IL-10 compared to DR3 mice after stimulation with LPS (Fig. 3D).

Furthermore, to determine whether *P. copri* induced tolerogenic dendritic cells (DCs) can promote regulatory T cell (Treg) differentiation, DCs were primed with either *P. copri* or control bacteria *D. piger* (known to promote proinflammatory response)*,* and co-cultured with naïve CD4⁺ T cells from DR3 mice in the presence of Treg-polarizing cytokines IL-2 and TGF-β. *P. copri*–primed DCs exhibited a trend toward increased CD4⁺CD25⁺FoxP3⁺ Treg differentiation compared with *D. piger*–primed controls (Fig. S4). These findings provide preliminary evidence that *P. copri* promotes Treg differentiation through DC-mediated immunomodulation, consistent with our in vivo observations. Thus, CD11c + DCs from DR3.IL-17A^−/−^ mice displayed a tolerogenic phenotype, marked by increased IL-10 production and trended reduced antigen presentation, that may affect disease severity. Overall, our data highlighted the role of gut microbiota (*Prevotella*)-Treg-IL-10 axis which might results in the modulation of disease in DR3.IL-17A^−/−^ mice.

### IL-17A deficient DR3 transgenic mice develop milder EAE disease

**Fig. 4:**
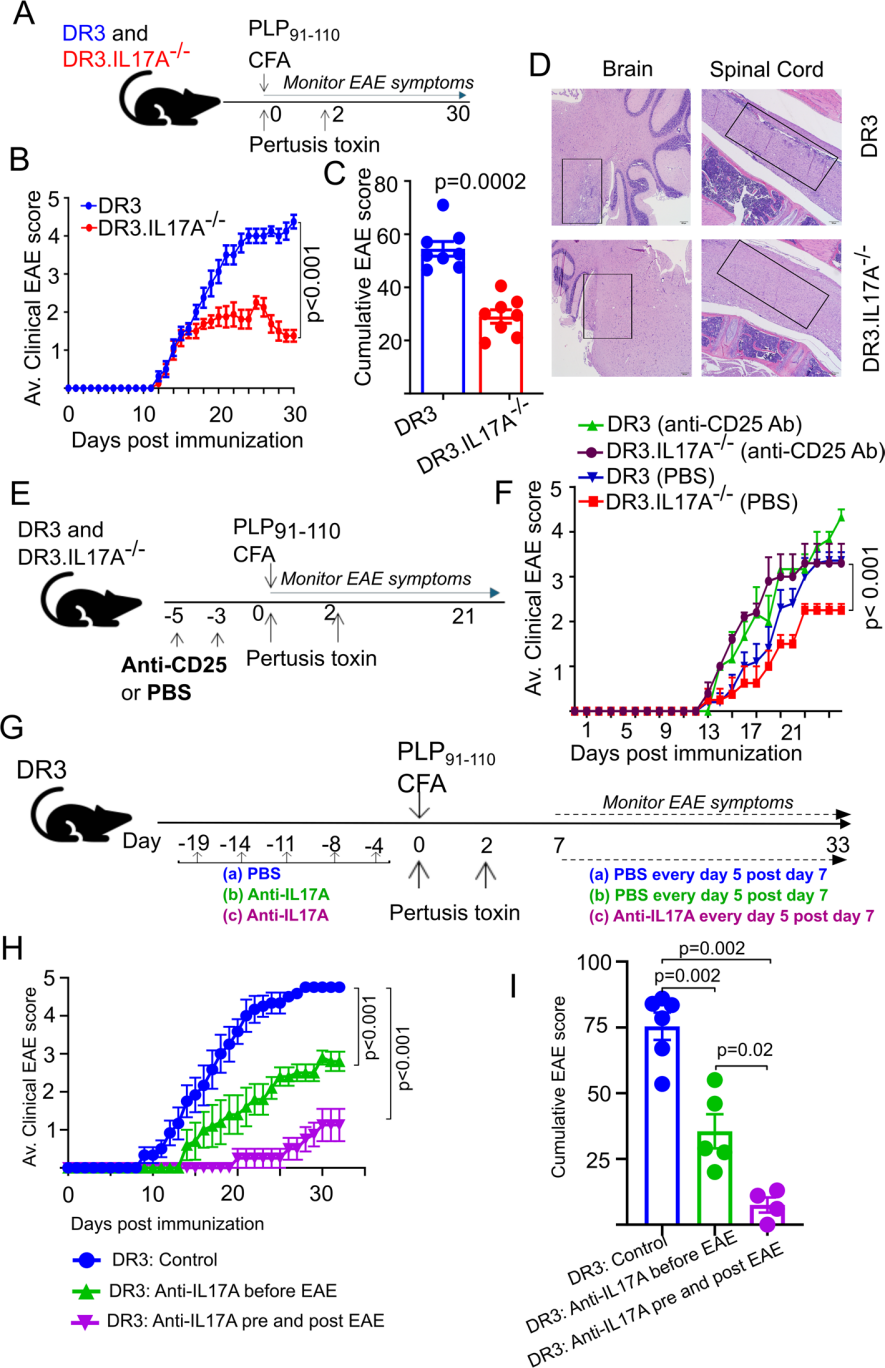
IL-17A-deficient DR3 mice (DR3.IL-17A^−/−^) exhibited ameliorated EAE compared to IL17A-sufficient DR3 mice. Eight to ten-week-old DR3 (n = 8) and DR3.IL-17A^−/−^ (n = 8) mice were immunized with PLP_91–110_ (50 ug) emulsified in CFA (200 ug). Pertussis toxin (80 ng) was administered *i.p* on day 0 and days 2. Mice were monitored daily. and EAE disease score were recorded until euthanizing on day 30 post-immunization. **A** Schematic representation of the experimental design for disease course in DR3 and DR3.IL-17A^−/−^ mice. **B** Average clinical EAE scores of DR3 and DR3.IL-17A^−/−^ mice over time. **C** Cumulative EAE scores from mice shown in B. **D** Representative histological images of CNS tissues from DR3 and DR3.IL-17A^−/−^ mice in B. **E** Schematic diagram showing the experimental design for DR3 and DR3.IL-17A^−/−^ mice treated with anti-CD25 to neutralize Treg prior to immunization with PLP_91–110_/CFA to induce EAE. Arrowheads represent days in which mice received anti-CD25 treatment. **F** Average clinical EAE scores of DR3 and DR3.IL-17A^−/−^ mice that were treated with anti-CD25 or PBS prior to induction of EAE. **G** Schematic representation of anti-IL-17A treatment in DR3 mice induced with EAE. Eight- to ten-week-old DR3 mice (n ≥ 4 mice per group) were treated with an anti-IL-17A monoclonal antibody before EAE induction with PLP_91–110/_CFA/PTX. **H** Average clinical EAE scores of mice treated as described in E. **I** Cumulative EAE scores of mice treated as described in E. Bars represent the mean and standard error of mean (SEM) in each group. Bullet points (B, F, H) or bars (C, I) represent the mean, and error bars indicates the SEM for each experimental group. *p*-value was determined using multiple unpaired t-tests (B, F), two-way ANOVA for EAE clinical scores (H), unpaired t-tests with Welch's correction (C), and ANOVA followed by pairwise comparisons (I). Data are representative of three independent experiments

As DR3.IL-17A^−/−^ mice showed enrichment of immunoregulatory pathways including increased frequency and function of Treg population, we hypothesized that in the absence of IL-17A (DR3.IL-17A^−/−^), mice would be resistant to EAE or develop attenuated disease. Thus, to test our hypothesis, we immunized both DR3 and DR3.IL-17A^−/−^ mice using PLP_91–110_ to induce EAE and monitored clinical symptoms every 24 h for 30 days (Fig. 4A). We observed that DR3.IL-17A^−/−^ mice developed significantly milder disease over time and had significantly lower cumulative clinical EAE scores compared to DR3 mice (Fig. 4B, C). Histological analysis of the brain and spinal cord tissues demonstrated decreased inflammatory cell infiltration in the brain and spinal cord (Fig. 4D). As the symptoms onset time in DR3 and DR3.IL-17A^−/−^ mice were similar and the disease was only different during the progression of the disease (Fig. 4B), our data hinted at no role of IL-17A for the disease induction but only during the disease progression.

The enrichment of *Prevotella* species in the gut microbiota, enhancement of FoxP3 and IL-10 by *P. copri* and subsequent reduction of EAE severity suggested toward *Prevotella*-Treg-IL-10 axis in regulating the immune response in DR3.IL-17A^−/−^ mice. Studies have shown that CD4^+^CD25^+^FoxP3^+^ Treg cells can regulate autoimmune inflammation in the body and suppress IL-17A-secreting CD4^+^ Th17 cells [40, 41]. To determine whether the increased Treg levels were responsible for milder disease phenotype in DR3.IL-17A^−/−^ mice, we treated these mice with an anti-CD25 blocking antibody to neutralize the Treg population before EAE induction (Fig. 4E) as described previously [42]. CD25 neutralization in DR3.IL-17A^−/−^ mice resulted in severe disease phenotype similar to DR3 transgenic mice, indicating that Treg were involved in establishing significantly lower disease severity in DR3.IL-17A^−/−^ mice (Fig. 4F).

First, to test if IL-17A plays no role in disease induction, we depleted IL-17A in DR3 mice by treating them with an anti-IL-17A antibody before and after induction of EAE (Fig. 4G). For the first group, mice were treated with the anti-IL-17A antibody on days −19, −14, −11, −8, and −4, before immunization. The second group of mice was treated with anti-IL-17A antibodies similar to the first group before immunization and also every five days after symptoms appeared in one of the groups after immunization. Compared to the control group treated with PBS both pre- and post-immunization, DR3 mice pre-treated with anti-IL-17A and administered PBS after disease induction still developed EAE but showed a significantly milder disease course, similar to that observed in DR3.IL-17A^−/−^ mice (Fig. 4H). However, DR3 mice treated with anti-IL-17A both before and after EAE showed a significant reduction in disease progression (Fig. 4H). The overall cumulative EAE clinical scores were also significantly lower in DR3 mice treated with anti-IL-17A pre- and post EAE induction compared to PBS-treated DR3 control and pre-EAE anti-IL-17A treated DR3 mice (Fig. 4I). Thus, our results indicate that IL-17A contributes to both the onset and progression of EAE, as anti-IL-17A treatment delayed disease development and reduced severity.

Next, to determine the role of IL-17A in disease progression and assess whether its effects extend beyond HLA-DR3 mice, we investigated the impact of IL-17A depletion in wild-type C57BL/6 J (B6) mice. B6 mice were immunized with MOG_35–55_ for EAE induction as described elsewhere [43]. Mice were then treated with PBS (control) or IgG1a (isotype control) or anti-IL-17A antibody on days 5, 8, 11, 14, 17, 20, 23, 26, 29, and 32 post-disease induction (Fig. S5A). Mice treated with anti-IL-17A post-EAE showed significantly lower disease severity over time compared to control and isotype control (Fig. S5B). The cumulative EAE scores were significantly lower in anti-IL-17A-antibody-treated mice compared to both control and isotype control (Fig. S5C) highlighting the role of IL-17A in disease progression. In a separate study, pre- and post-EAE treatment of the B6 mice with anti-IL-17A antibody resulted in significantly lower disease severity and overall cumulative EAE scores compared to PBS or IgG1a-treated mice (Fig. S5D, S5E, S5F). These data indicated that the IL-17A is redundant for disease induction but crucial in disease progression in both PLP_91–110_-DR3-EAE and MOG_35–55_-B6-EAE models.

### Neither IL-17F nor GM-CSF compensate for disease in IL-17A deficient DR3 transgenic mice

Even though IL-17A is considered an important cytokine for the development of EAE [44], our results suggest that IL-17A is crucial for disease progression. In this case, the induction of EAE may be compensated by IL-17F cytokine which shares 55% homology at the amino acid level with IL-17A [45]. Although, DR3.IL-17A^−/−^ mice have significantly higher levels of IL-17F compared to IL-17A sufficient DR3 mice (Fig. S6), neutralizing IL-17F did not affect the disease onset and severity in DR3.IL-17A^−/−^ mice compared to isotype control IgGa1-treated mice (Fig. S7A, S7B). Also, GM-CSF has been previously shown to be a pathogenic cytokine in EAE [46]. The neutralization of the GM-CSF in DR3.IL-17A^−/−^ mice had no effect on disease onset but resulted in an increased average disease severity over time (Fig. S7A, S7B). Thus, niether GM-CSF nor IL-17F could compensate for the IL-17A in DR3.IL-17A^−/−^ mice for disease induction, and our data indicate that IL-17A, IL-17F, and GM-CSF are not required for the development of EAE disease.

### *Prevotella*-related species are transferred from IL-17A deficient to IL-17A sufficient mice

**Fig. 5:**
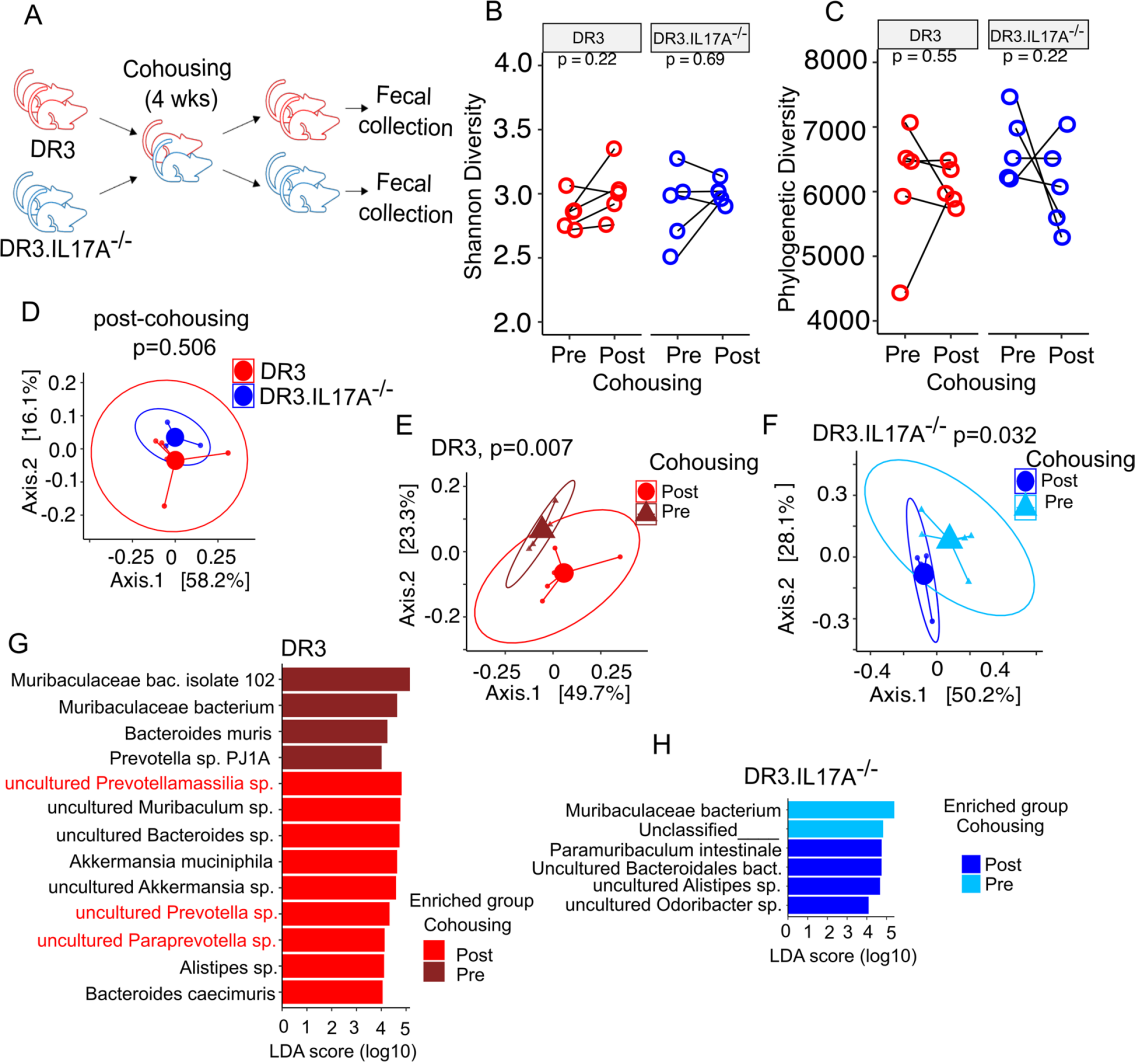
*Prevotella*-related species are transferred to DR3 mice from DR3.IL-17A^−/−^ mice when DR3 and DR3.IL-17A^−/−^ mice were cohoused. Eight to ten-week-old DR3 (n = 5) and DR3.IL-17A^−/−^ (n = 5) mice fecal samples were collected. Fecal from DR3 mice was transplanted to DR3.IL-17A^−/−^ mice, and conversely, fecal materials from DR3.IL-17A^−/−^ mice was transplanted into DR3 mice via oral gavage. Five days after oral gavage, DR3 mice were cohoused with DR3.IL-17A^−/−^ mice. Fecal samples were collected before and after co-housing/fecal transplant for shotgun metagenomic sequencing. **A** Schematic figure of the experiment. **B** Shannon and (**C**) Phylogenetic diversity in DR3 and DR3.IL-17A^−/−^ mice pre- and post-cohousing. **D** Bray–Curtis dissimilarity metric-based PCoA plots showing microbiome composition in DR3 and DR3.IL-17A^−/−^ post cohousing microbiome. **E** DR3 pre- and post-cohousing microbiome (**F**) DR3.IL-17A^−/−^ pre- and post-cohousing microbiome. **G** Differential abundance of bacterial species using *lefse* from *microbiomeMarker* package at *kw_cutoff* = 0.01, *wilcoxon_cutoff* = 0.01 and *lda_cutoff* = 4 in R for DR3 mice pre- and post-cohousing and (**H**) Differential abundance of bacterial species for DR3.IL-17A^−/−^ mice pre- and post-cohousing. Unpaired Wilcoxon-test was performed for B and C *adonis2* test was performed for D, E, F

Next, we investigated whether that transferring the gut microbiome from DR3.IL-17A^−/−^ mice to DR3 mice also led to transfer of *Prevotella* species ultimately affecting Treg frequency and EAE severity. Thus, we co-housed DR3 mice with DR3.IL-17A^−/−^ mice for four weeks and collected fecal samples (Fig. 5A). First, we analyzed the gut microbiome of DR3 and DR3.IL-17A^−/−^ mice pre- and four weeks post-cohousing. No differences in the alpha diversity was observed for both DR3 and DR3.IL-17A^−/−^ mice pre- vs post-cohousing as measured by Shannon diversity and phylogenetic diversity (Fig. 5B, C). Also, the gut microbiome of DR3 and DR3.IL-17A^−/−^ was compositionally similar (Fig. 5D) and no significantly altering bacteria were observed between the two groups. However, when comparing pre- vs post-cohousing, there was significant alteration of the gut microbiome composition for both DR3 and DR3.IL-17A^−/−^ mice (Fig. 5E, F). Thus, even though the gut microbiome was different between the DR3 and DR3.IL-17A^−/−^ mice prior to cohousing (Fig. 1D, E), it significantly changed after cohousing for DR3 and DR3.IL-17A^−/−^ mice for each group but converged to become similar post-cohousing. Functionally, after fecal transplant followed by cohousing, there was no differences in the functional pathways between DR3 and DR3.IL-17A^−/−^ mice (Fig. S8A). Interestingly, gut microbiome of DR3 mice altered subtantially when comparing pre and post cohousing microbiome (Fig. S8B). Using LEfSe analysis, we identified 23 differentially enriched pathways in DR3 mice between the pre and post cohousing. Majority of metabolic pathways (21 out of 23) were enriched after cohousing of DR3 with DR3.IL-17A^−/−^ mice (Fig. S8D) suggesting the gut microbial genetic enrichment of DR3 mice after cohousing. In contrast, no significant differences were observed in the microbiome of DR3.IL-17A^−/−^ mice pre- and post cohousing (Fig. S8C). Together, these findings suggest that co-housing altered the microbiome composition of DR3 mice, enriching their metabolic potential and making their microbial community more similar to that of DR3.IL-17A^−/−^ mice, likely through transfer of specific gut bacterial species from DR3.IL-17A^−/−^ to DR3 mice. Interestingly, differential abundance of the gut microbiome taxonomy revealed that DR3 mice acquired multiple *Prevotella* species that were present in DR3.IL-17A^−/−^ mice such as uncultured *Prevotellamassilia* sp., uncultured *Prevotella* sp. and uncultured *Paraprevotella* sp. (Fig. 5G). However, we observed bacteria such as *Paramuribaculum intestinale* and uncultured *Bacteroidales* bacterium being transferred to DR3.IL-17A^−/−^ mice (Fig. 5H). These data suggest that *Prevotella* species that were transferred from DR3.IL-17A^−/−^ mice may be critical in transferring Treg modulatory phenotype in the gut to ultimately affect disease severity in DR3 mice.

### *Prevotella*-related species’ Treg proliferating phenotype is transferred from IL-17A deficient to IL-17A sufficient mice ameliorated EAE severity

**Fig. 6:**
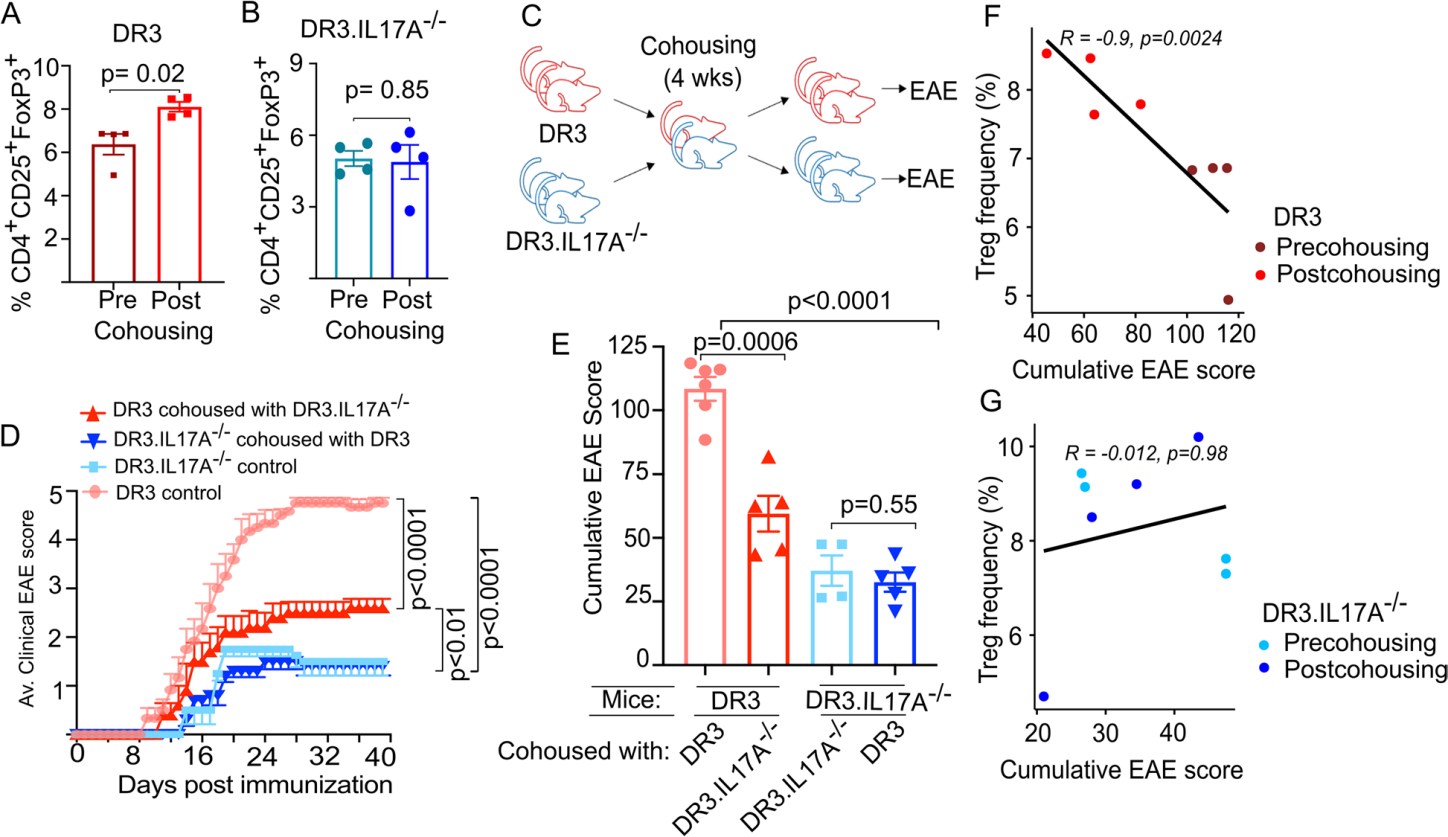
Tregs transferred from DR3.IL-17A^−/−^ to DR3 when cohoused together results in lower disease severity in DR3. 8–10-week-old female DR3 mice were cohoused for four weeks with DR3.IL-17A^−/−^ mice after one-time fecal transferred (orally gavaged) from each other mice strains. Peripheral blood mononuclear cells (PBMCs) were isolated from blood that was collected by retro-orbital bleeding, and Treg cells were assessed by flow cytometric analysis. **A**-**B** Percentages of CD4^+^CD25^+^FoxP3^+^ Treg cell populations in DR3 and DR3.IL-17A^−/−^ mice pre- and post-cohousing. **C** Schematic of experimental flow for cohousing of DR3 and DR3.IL-17A^−/−^ mice. **D** Average clinical EAE scores of DR3 mice (non-cohoused control and after cohoused with DR3.IL-17A^−/−^ mice) and DR3.IL-17A^−/−^ mice (non-cohoused control and after cohoused with DR3 mice) that were immunized with PLP_91–110_/CFA and followed for 30 days. **E** Average cumulative EAE scores of mice in D. **F**-**G** Spearman correlation of percentage of Tregs in blood to cumulative EAE scores in DR3 and DR3.IL-17A^−/−^ mice. Unpaired Wilcoxon-test was performed for A, B. Data represent the mean and standard error of the mean for each experimental group from two independent experiments: n 4–6 mice per group. *p*-value was determined by two-way ANOVA for clinical EAE scores

Next, to investigate if Treg cells modulatory phenotype is transferred to DR3 mice from DR3.IL-17A^−/−^ mice after cohousing for four weeks, we assessed the levels of Treg in the peripheral blood for both IL-17A deficient and IL-17A sufficient mice pre- and post-cohousing. Indeed, we observed that CD4^+^CD25^+^FoxP3^+^ Treg cell populations were significantly increased in DR3 mice after cohousing with DR3.IL-17A^−/−^ mice in peripheral blood (Fig. 6A). In contrast, Treg populations were not different in DR3.IL-17A^−/−^ mice post-cohousing with DR3 mice (Fig. 6B).

Finally, we examined whether cohousing-facilitated *Prevotella*-related species transfer and enhanced Treg proliferation altered EAE severity in DR3 mice. For disease induction, after cohousing DR3 and DR3.IL-17A^−/−^ mice for four weeks, they were housed separately (Fig. 6C). Post-cohousing, DR3 mice showed significantly reduced disease severity compared to non-cohoused DR3 control counterparts (Fig. 6D). The significantly lower disease severity in non-cohoused control DR3.IL-17A^−/−^ compared to non-cohoused DR3 mice was intact. However, post-cohoused DR3.IL-17A^−/−^ mice and non-cohoused DR3.IL-17A^−/−^ mice had similar lower disease severity phenotype (Fig. 6D). In addition, DR3 mice cohoused with DR3.IL-17A^−/−^ mice had significant reduction in cumulative EAE scores but no differences in cumulative EAE scores was observed for DR3.IL-17A^−/−^ mice when cohoused with DR3 (Fig. 6E). Thus, our data implied that the microbiome transfer from DR3.IL-17A^−/−^ mice to DR3 post cohousing had significant effect on disease severity but not vice-versa. Also, we observed a significant negative correlation of Tregs with cumumative EAE scores in DR3 mice pre- and post-cohousing while no such significant correlation was observed for the DR3.IL-17A^−/−^ mice (Fig. 6F, G). These data suggest that lack of IL-17A altered the gut microbiome composition to enrich Treg-proliferating *Prevotella*-related species; the transfer of which was critical in reducing the overall disesase severity in to DR3 mice.

**Fig. 7:**
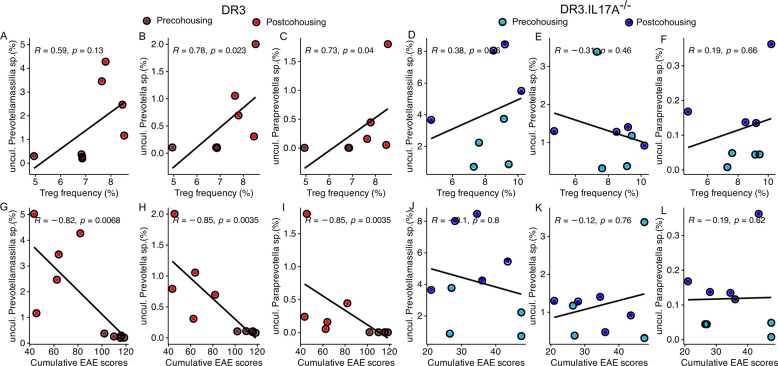
Spearman correlation of *Prevotella*-related species with Treg population (%) and cumulative EAE scores in DR3 and DR3.IL-17A^−/−^ mice pre- and post-cohousing. R and p represent coefficient of correlation and p-value respectively

To confirm that the *Prevotella*-related species were critical in reduction of disease severity, we correlated the abundances of differentially abundant *Prevotella*-related species in DR3 mice after cohousing with DR3.IL-17A^−/−^ mice (Fig. 6G) to Treg frequency and cumulative EAE scores in both DR3 and DR3.IL-17A^−/−^ mice pre- and post cohousing (Fig. 7). Interestingly, we observed that uncultured *Prevotella* sp (R = 0.78, p = 0.023) and *Paraprevotella* sps (R = 0.73, p = 0.04) but not *Prevotellamassilia* sp. (R = 0.59, p = 0.13), was significantly positively correlated with the peripheral Treg levels in DR3 mice while no significant correlation was observed for all three bacteria in DR3.IL-17A^−/−^ mice (Fig. 7A, B, C, D, E, F). However, above mentioned three bacteria were significantly negatively correlated with cumulative EAE scores in DR3 mice and no such correlation was observed in DR3.IL-17A^−/−^ mice (Fig. 7G, H, I, J, K, L). These data suggest that microbes transferred from DR3.IL-17A^−/−^ mice to DR3 mice such as *Prevotella*-related species specifically, *Prevotella* sp and *Paraprevotella* sp. were crucial in enhancing the Treg cells population and ultimately reducing EAE severity in DR3 mice.

## Discussion

This study highlights a previously underappreciated role for IL-17A in regulating the gut microbiota, mucosal immunity, and CNS autoimmunity using a humanized HLA-DR3 transgenic model of MS. IL-17A deficiency resulted in a distinct gut microbial composition characterized by altered microbiota specially enrichment of *Prevotella* species, upregulated PPAR signaling (a pathway that can also induce Treg differentiation), and expansion of the Treg population with enhanced Treg function. The administration of commensal *Prevotella* species, *P. copri* further validated its role in promoting immunomodulatory response. These changes were accompanied by elevated IL-10 production and a significant reduction in the severity of EAE. Importantly, these features were transferable via FMT and cohousing, demonstrating that a microbiota-driven regulatory circuit, rather than developmental absence of IL-17A alone, underlies the protective phenotype.

While a prior study have reported association of gut microbiota in IL-17A deficneint mice [19], they did not report any significant differences in Treg populations between IL-17A deficient and wild-type mice. In contrast, our study showed an increase in Tregs in DR3.IL-17A^−/−^ mice, which appears to be directly linked to gut microbiota changes specifically abundance of *Prevotella species*. This discrepancy may stem from differences in the genetic background and facility enviornment. In gut, lack of IL-17A alone enriched *Prevotella*-related species in DR3.IL-17A^−/−^ mice and suggested an inverse relation between IL-17A and *Prevotella* abundance. Intrestingly, a human MS study had also reported an inverse correlation between the abundance of *Prevotella* in mucosal biopsies and IL-17A^+^ CD4^+^ T cells, as well as disease severity [47]. Specifically, MS patients with severe disease and elevated IL-17A^+^ CD4^+^ T cells exhibited reduced *Prevotella* abundance in mucosal biopsies. These findings underscore the reciprocal relationship between IL-17A signaling and *Prevotella*, linking cytokine-microbiota interactions with immune modulation in both human and mouse settings.

Transcriptomic and protein analyses confirmed PPAR-γ pathway activation (PPARγ, PLIN1) in IL-17A-deficient DR3 mice. Genes belonging to PPAR signaling categories are indicated as important mediators of immunoregulatory responses [48, 49]. The concurrent enrichment of *Prevotella* species and activation of the PPAR-Treg axis in DR3.IL-17A^−/−^ mice suggests a compensatory anti-inflammatory mechanism. IL-17A contributes to intestinal homeostasis through the maintenance of epithelial barrier integrity and regulation of antimicrobial peptide expression [50, 51]. In its absence, altered mucosal immunity and nutrient microenvironments may favor expansion of fiber metabolziizng taxa such as *Prevotella*, which were enriched in DR3.IL-17A^−/−^ mice. Beyond barrier effects, IL-17A deficiency can also reshape the metabolic landscape of the gut by reducing inflammation-driven oxygenation and reactive nitrogen species, creating an anaerobic niche conducive to *Prevotella* and other SCFA-producing commensals [52, 53]. These commensals generate metabolites such as acetate, propionate, and butyrate that activate PPAR signaling in epithelial and immune cells [54], promoting FoxP3⁺ Treg differentiation and IL-10 production [55]. The fact that these features were transferable by FMT and cohousing supports a microbiota-driven mechanism, wherein IL-17A deficiency indirectly enhances a regulatory pathway involving *Prevotella*-derived metabolites, PPAR activation, Treg differentiation, and IL-10 induction, collectively leading to attenuation of neuroinflammation. Imporatnce of *Prevotella* species in induction of Treg related genes and pathways was further confirmed by administering *P. copri* (DSM 18205) treatment to DR3 mice. This treatment enriched colonic PPAR pathway genes and, importantly, also increased FoxP3 and IL-10 gene expression, indicating that *P. copri* exerts immunoregulatory effect in the colon by enhancing PPAR signaling linked to Treg and IL-10 induction. In fact, the gut microbiome-induction of Treg in IL-17A deficient mice was concurrent with the increased production of IL-10 from splenic DCs from DR3.IL-17A^−/−^ mice. It suggests that the tolerogenic DCs are induced in the absence of IL-17A, and DC-derived IL-10 is essential to ameliorate EAE severity [56]. The reduced trends of antigen presentation capacity of DCs from DR3.IL-17A^−/−^ mice further support the role of gut microbiota-induced tolerogenic DCs in mice lacking IL-17A. SCFAs such as propionate and butyrate have been demonstrated as potent inducers of tolerogenic DCs [57] and thus, SCFAs produced by the gut microbiota like *Prevotella* species in DR3.IL-17A^−/−^ mice can induce tolerogenic DCs in IL-17A deficient mice.

Consistent with these findings, our recently published human microbiome study [38] provided direct experimental evidence supporting the protective role of *P. copri* in CNS autoimmunity. In that study, microbiota-depleted mice were first mono-colonized with human-derived *P. copri, a* taxa enriched in healthy controls or *Blautia wexlerae*, a bacteria enriched in MS patients, and subsequently reconstituted with a full healthy human microbiota. Mice colonized with the *P. copri* exhibited enhanced anti-inflammatory responses and developed markedly milder EAE compared with those harboring MS-associated bacteria [38].

These data reinforce our current observation that *Prevotella* enrichment promotes a PPAR-Treg-IL-10–associated immunoregulatory milieu, highlighting a consistent microbiota–immune axis across both human and humanized mouse models of MS. While these findings suggest a link between *Prevotella*-driven metabolic signaling and Treg-mediated suppression of inflammation, additional mechanistic studies are needed to determine whether PPAR activation directly mediates the protective effects observed in this model.

In addition to the enrichment of *Prevotella* species, uncultured *Muribaculum* species were also highly represented in the gut microbiota of DR3.IL-17A^−/−^ mice. Members of the Muribaculaceae family are abundant commensals in murine intestines and have been associated with SCFA production, including propionate and succinate, which can influence host lipid metabolism and PPAR signaling [58, 59]. Such metabolites may contribute to the observed induction of PPAR-dependent pathways and the expansion of regulatory T cells in DR3.IL-17A^−/−^ mice. Although *Muribaculum* species are not well characterized in humans, their metabolic potential suggests they may act synergistically with *Prevotella* to promote an anti-inflammatory mucosal environment.

Although our data point toward an expansion of the Treg population and its contribution to the milder disease observed in DR3.IL-17A⁻^/^⁻ mice, it is possible that additional mechanisms beyond Tregs and IL-10 also play a role in disease attenuation. The absence of IL-17A likely reduces the pool of pathogenic Th17 cells, dampens pro-inflammatory cytokine signaling, and weakens antigen-presenting cell activation, collectively leading to reduced immune activation. Thus, the milder disease in DR3.IL-17A⁻^/^⁻ mice likely reflects a combination of diminished Th17-driven inflammation and enhanced Treg-mediated regulation. Future mechanistic dissection of Treg expansion, including transcriptomic or epigenetic profiling of isolated Tregs, will help define molecular mechanisms underlying their enhanced function. Additionally, detailed analysis of Th17 pathogenicity, such as cytokine profiling, transcription factor expression, and metabolic characterization, will be important to fully understand the immunoregulatory balance in this model.

The enrichment of immunoregulatory pathways in DR3.IL-17A^−/−^ mice was accompanied by a significantly attenuated EAE disease, supporting prior reports that loss of IL-17A reduces EAE severity [19]. Our data extend these findings by demonstrating that IL-17A deficiency reshapes the gut microbiota, particularly enriching *Prevotella* species, which promotes PPAR signaling, Treg differentiation, and IL-10 production. Differences between our findings and those reported by Regan et al. [19] may reflect dissimilar genetic background: (C57BL/6 J vs HLA-DR3), the use of IL-17^−/−^ vs IL-17A^−/−^ models, and animal facility environment.

We have also previously shown that introducing *Prevotella* species into the gut can alleviate EAE severity through changes in the gut microbiota that ultimately promote Treg development [60, 61]. The cohousing experiments further validated that these microbiota-driven regulatory effects are transmissible: exposure of IL-17A-sufficient DR3 mice to the gut microbiota of IL-17A-deficient mice resulted in *Prevotella* enrichment, Treg induction, and disease attenuation. These results demonstrate that Treg-promoting microbiota are dominant over disease-promoting communities and can transfer protective effects to genetically susceptible hosts, providing a strong rationale for exploring microbiota-based therapeutic interventions in MS.

DR3.IL-17A^−/−^ mice showed increased CD4⁺CD25⁺FoxP3⁺ Tregs, and disease protection was reversed by anti-CD25 (PC61)–mediated depletion, aligning with similar observations in GM-CSF-deficient mice [62]. Thus, these findings suggest that pathogenic cytokines such as IL-17A and GM-CSF may promote EAE progression by suppressing regulatory CD4⁺CD25⁺ T cells. However, since anti-CD25–mediated depletion is not specific to Tregs and may also target activated effector T cells, these results should be interpreted with caution. Future studies employing Foxp3-DTR mice or other selective Treg depletion models will be required to precisely define the regulatory role of Tregs in modulating disease severity in the absence of IL-17A.

It is possible that other Th17 cytokines (IL-17F and GM-CSF) can compensate for IL-17A loss for disease development. However, blockade of either IL-17F or GM-CSF did not prevent induction in our model, consistent with reports showing redundancy or disease-phase-specific roles for these mediators [46, 63–65]. Thus, our data suggest that IL-17A, together with IL-17F, or GM-CSF is dispensable for EAE disease.

The clinical relevance of these findings is further supported by multiple human studies linking gut microbiome composition and HLA genotype with immune dysregulation in MS. *Prevotella* species, including *P. copri* is consistently reported to be depleted in MS cohorts, and their reduced abundance correlates with elevated IL-17A-producing T cells and increased disease activity [38, 61]. Our data using HLA-DR3 transgenic mice, representing a key MS susceptibility allele, demonstrate that IL-17A deficiency promotes *Prevotella* enrichment and up-regulates the PPAR-Treg-IL-10 axis, mirroring the immunoregulatory patterns seen in healthy individuals. These results provide a mechanistic bridge between genetic predisposition and microbial modulation of mucosal immunity in MS. Together with prior human studies, our work supports the translational potential of microbiota-targeted therapies, such as *Prevotella*-based probiotics or combination regimens integrating anti-IL-17A therapy with microbiome restoration, to improve immune balance and clinical outcomes in MS.

In summary, our study highlights an important pathway through which IL-17A regulates inflammation, specifically by modulating the gut microbiota to suppress pathways linked with Treg expansion and function. Our study point towards a mechanism by which immune mediators such as cytokines impact the gut microbiota to alter immune cell function and, ultimately, disease outcomes. Our results have strong clinical relevance as modulation of the gut microbiota are being considered as potential therapeutics to treat diseases ranging from inflammatory to neurological diseases. As MS is characterized by both inflammation and gut dysbiosis, combination therapies targeted to reduce inflammation as well as correct gut dysbiosis might result in better therapeutic outcome than either one alone. Although IL-17A had been linked with the pathobiology of MS, two anti-IL-17A clinical trials in MS (NCT01874340 and NCT01433250) were terminated early. In that regard, while anti-IL-17A therapy alone was not as effective as current MS drugs, a combination of anti-IL-17A and microbiota modulation might provide better therapeutic efficacy. Additionally, Treg induction is also being tried as a potential treatment option in MS [66]. Finally, our FMT plus co-housing experiments emphasize that gut commensals capable of inducing Tregs may not only correct dysbiosis but also reshape systemic immunity. Future studies exploring defined Treg-inducing commensals in combination with anti-inflammatory therapies hold promise for advancing precision microbiota-based treatments in MS.

## Methods

### Study design

The study utilized female HLA-DR3 transgenic mice, HLA-DR3.IL-17A^−/−^ transgenic mice, and C57BL/6 J mice to investigate the contributions of IL-17A to EAE. The disease was induced by immunizing mice with the PLP_91–110_ peptide in HLA-DR3 transgenic mice, HLA-DR3.IL-17A^−/−^ transgenic mice. C57BL/6 mice were immunized with MOG_35−55_ peptide. The disease induction and scoring of the EAE was performed as described below and elsewhere [67]. No data, including outliers, were excluded from the analysis. Experiments were performed at least twice with some exceptions as indicated in the Figure legends.

### Mouse procurement and rearing

HLA-DR3 (DRA1*0101, DRB1*0301) transgenic mice on the B6/129 background have been characterized previously [25, 68]. These mice lack endogenous murine major histocompatibility complex (MHC) class II genes (AE^−/−^) and express alleles DRA1*0101, DRB1*0301, as described previously [25, 68]. The HLA-DR3.IL-17A^−/−^ mice (referred to as DR3.IL-17A^−/−^) that were used in this study were generated by crossing HLA-DR3 (referred to as DR3) and AE^−/−^.IL17A^−/−^ mice and validated using genotyping. C57BL/6 mice were also utilized in this study and were purchased from the Jackson Laboratories (Bar Harbor, ME). Female DR3 mice, DR3.IL-17A^−/−^ mice, and C57BL/6 mice (8–10 weeks of age) were used in this study. Mice were bred and maintained in the University of Iowa animal facility in accordance with NIH and institutional guidelines. All experiments were approved by the Institutional Animal Care and Use Committee at the University of Iowa.

### Microbiota DNA isolation, sequencing, and analysis

8–10 week old DR3 and DR3.IL-17A^−/−^ female mice were assessed for the composition of their gut microbiota using in-depth shotgun metagenomic sequencing on the fecal DNA, which provides species-level taxonomic resolution and enables functional pathway analysis. DNA was extracted using PowerSoil DNA Isolation Kit (Qiagen) following the manufacturer’s protocol. Each metagenomic DNA was quantified and sequenced using an Illumina MiSeq. Relative abundances of genes and pathways were used for downstream statistical analysis. Statistical significance between the groups were determined using non-parametric Wilcoxon signed rank test, and data was visualized using GraphPad Prism.

Each metagenomic DNA was quantified and sequenced using an Illumina MiSeq resulting in more than 55 Gb of raw paired-end reads from 20 samples after shotgun metagenomic sequencing. The raw paired-end metagenomes were quality trimmed using Trim Galore version 0.5.0, followed by host read filtering by mapping to the mouse genome (GRCm39) using Bowtie2 [69], with default parameters resulting in 49.43 Gb of high-quality data. The quality trimmed and filtered forward and reverse reads were taxonomically characterized using *kaiju* 1.10.1 [70] in greedy mode. Reads were searched against the *proGenomes* reference database of protein sequences (downloaded, June 3, 2021) [71]. Further downstream analysis was conducted using phyloseq package in R 4.0.3 [72]. Shannon entropy and phylogenetic diversity were calculated and plotted using *ggplot2.* Bacterial taxa at the species level not observed at least 100 times in a minimum of 10% of the samples were removed and the samples were normalized using median read counts. *Beta-*diversity was measured using Bray–Curtis dissimilarity metric. For differential analysis, *run_lefse* function was used from the *microbiotaprocess* package in R with parameters as stated in individual figure legends. HUMANn3 [73] was used with default parameters to profile functional pathway abundances in the clean reads using MetaCyC databases. Unstratified pathways that were not observed at least 10 times in at least 10% of the samples were removed, normalized to median depth, and analyzed downstream in R. Bray–Curtis dissimilarity metrics was used to differentiate the functions of the microbiota using PCoA plots. For differential analysis, run_lefse function was used from the microbiotaprocess package in R with parameters as stated in individual figure legends. All statistical analysis were conducted as stated in the individual figure legends.

### Transcriptome sequencing and analysis

Tissue sections from the colon of both DR3.IL-17A^−/−^ and DR3 mice before EAE induction were collected, chopped into small pieces, stored in RNAlater (Millipore Sigma, MO, USA), and frozen to −80 °C until further use. RNA was extracted using the RNeasy Kit (Qiagen) following the manufacturer’s instructions. The quality and quantity of the RNA was evaluated using a NanoDrop and sequenced at Novogene Co. Ltd, CA. Sequences were quality controlled using default *fastqc* and paired FASTQ files were aligned to the mm10 genome build with the kallisto pseudoaligner [74] using 100 bootstraps. Estimated counts for transcripts were then aggregated into gene-level quantifications based on gene symbol. An average of 14.79 ± 1.3 million transcripts were obtained for each sample. Further downstream processes were carried out in R 4.0.3 [72] using edgeR v3.32.1 [75] to identify differentially expressed genes, as described previously. Differentially expressed genes were fed into ShinyGO [76] to identify changes to genes involved in KEGG database using the mouse reference database.

### Quantification of PPAR signaling pathways genes using qPCR

Tissue sections from the colon of 8-weeks old DR3.IL-17A^−/−^ and DR3 mice were collected, and RNA was extracted using the RNeasy Kit (Qiagen) following the manufacturer’s instructions. The quality and quantity of the RNA was evaluated using a NanoDrop. To determine the expression of PPAR signaling pathway genes, namely *Adipoq*, *Plin1*, *Plin4*, and *Fabp4*, a two-step quantitative reverse transcriptase-mediated real-time PCR (qPCR) was performed. Equal aliquots of total RNA (2 µg) from colonic samples of DR3.IL-17A^−/−^ (n = 5) and DR3 mice (n = 5) were reverse transcribed to cDNA using the High-Capacity iScript cDNA synthesis kit (Bio-Rad). qPCR reactions were then carried out in triplicate using 50 ng of cDNA and the Applied Biosystems Power SYBR Green PCR Master Mix (Applied Biosystems). Amplification data were collected using an Applied Biosystems Prism 7900 sequence detector and analyzed with Sequence Detection System software (Applied Biosystems). The qPCR primers for the genes were purchased from Integrated DNA Technologies, Inc. The primer sequences used were as follows:

*Adipoq*: Fwd: AGATGGCACTCCTGGAGAGAAG, Rev: ACATAAGCGGCTTCTCCAGGCT

*Plin1*: Fwd: GAGAAGGTGGTAGAGTTCCTCC, Rev: GTGTGTCGAGAAAGAGTGTTGGC

*Plin4*: Fwd: GCACTAAGGACACGGTGACCAC, Rev: GACCACAGACTTGGTAGTGTCC

*Fabp4*: Fwd: TGAAATCACCGCAGACGACAGG, Rev: GCTTGTCACCATCTCGTTTTCTC

The data obtained from qPCR were normalized to the expression of the housekeeping gene GAPDH.

To assess the effect of *Prevotella copri* (*P. copri* DSM 18205) treatment on the expression of PPAR signaling pathway genes, DR3 mice were divided into two groups. One group of mice was treated with *P. copri*, while the other group was treated with BHI media (control). The *P. copri* group received 10^7^ CFUs of live *P. copri* DSM 18205 via oral gavage every other day for a total of seven doses. The control group received 200 µl of BHI media alone by oral gavage. Tissue sections from the colon of *P. copri*-treated and BHI media-treated DR3 mice were collected, and RNA extraction and qPCR analysis for the expression of PPAR signaling pathway genes (*Adipoq, Plin1, Plin4,* and *Fabp4*), Treg, and IL10 genes were performed as described above.

### Western blot analysis (PPARγ)

Fresh colon tissues from DR3 and DR3.IL-17A^−/−^ mice were lysed in RIPA buffer supplemented with cOmplete Protease Inhibitor Cocktail (EDTA-free) and PhosSTOP Phosphatase Inhibitor Cocktail (Roche). Total protein concentrations were determined using the Pierce™ BCA Protein Assay Kit (Thermo Fisher Scientific, Waltham, MA, USA). Equal amounts (50 µg) of protein were resolved on 4–15% SDS–PAGE gradient gels (Bio-Rad, Hercules, CA, USA) and transferred to PVDF membranes. Membranes were blocked for 1 h with 5% non-fat milk in TBST, incubated overnight with primary antibodies, washed (3 × 5 min, TBST), then incubated with horseradish peroxidase (HRP)-conjugated secondary antibodies, washed again, and developed using enhanced chemiluminescence. The following antibodies were used: Anti-PPARγ (1:1000; D69, #2430, RRID: AB_823599) and Anti-Perilipin 1 (1:1000; D1D8, #9349, RRID: AB_10829911) from Cell Signaling Technology (Danvers, MA, USA), and Anti-β-actin (1:10,000; AC-15, #A544, RRID: AB_4767441) from Sigma-Aldrich (St. Louis, MO, USA). HRP-conjugated secondary antibodies-anti-mouse IgG-HRP (#sc-2055, RRID: AB_631738) and anti-rabbit IgG-HRP (#sc-2054, RRID: AB_631748) were obtained from Santa Cruz Biotechnology and used at 1:5000 for PPARγ and Perilipin 1 or 1:10,000 for β-actin.

### EAE induction, scoring and monitoring

To induce EAE, DR3 and DR3.IL-17A^−/−^ mice (8 to 12 weeks old) were immunized subcutaneously in both flanks with 50 µg of PLP_91–110_ (YTTGAVRQIFG DYKTTICGK) (GenScript, Piscataway NJ, USA) that was emulsified in CFA containing 200 μg/mouse *Mycobacterium tuberculosis* H37Ra (Becton, Dickinson and Company, Sparks, MD, USA). 80 ng of pertussis toxin (PTX) (Sigma Chemicals, St. Louis, MO) was given intraperitoneal at day 0 and 2 post immunization. C57BL/6 mice were immunized subcutaneously in both flanks with MOG_35–55_ (MEVGWYRSPFSRVVHLYRNGK) that was emulsified in CFA. PTX was given as described for immunization with PLP [77]. Mice were observed daily for disease symptoms and EAE was scored with the following scoring system, as described previously [67]: normal, 0; loss of tail tone, 1; hind limb weakness, 2; hind limb paralysis, 3; hind limb paralysis and forelimb paralysis or weakness, 4; and morbidity/death, 5.

### Pathology

Brains and spinal cords were collected from DR3, and DR3.IL-17A^−/−^ mice at day 30 post immunization. Histological analysis was performed to assess inflammation and demyelination, as described previously [25, 78]. Briefly, DR3 mice and DR3.IL-17A^−/−^ mice were perfused with approximately 50 ml of 10% neutral buffered formalin via intracardiac puncture. The calvaria and spinal columns were collected, and immersion fixed in 10% neutral buffered formalin (10% BFA) for 24–48 h followed by decalcification in EDTA for approximately 24 h. Decalcified tissues were cut into 2 mm coronal blocks, embedded in paraffin and routinely processed. The slides were cut at 4–5 mm thickness and stained with hematoxylin and eosin. HE-stained slides including the brainstem, cortex, corpus callosum, cerebellum, hippocampus, and striatum were examined and scored by a board-certified veterinary pathologist as described previously [25, 78].

### Flow cytometry

Peripheral blood mononuclear cells (PBMCs) were isolated from naïve DR3 and DR3.IL-17A^−/−^ mice using Histopaque-1077 (Millipore Sigma, USA) density centrifugation method as per instructions from the manufacturer (Sigma-Aldrich, St. Louis, USA). Briefly, 200 µl of blood was collected in an EDTA-containing Eppendorf tube by retroorbital bleeding. Blood was diluted with 800 ml of phosphate-buffered saline (PBS; Gibco/Lubioscience) and added to 1 ml of Histopaque-1077, and then centrifuged at 400 × g for 30 min at room temperature, without the brake. Following centrifugation, the buffy coat was collected into 5 mL tubes and washed with PBS (400 × g for 10 min at 4 °C). The cells were washed with FACS buffer stained with antibodies to detect surface expression of CD4 (GK1.5) and CD25 (PC61) (BD Biosciences, Franklin Lakes, NJ), whereas intracellular expression of FoxP3^+^ was stained using an anti-Mouse/Rat FoxP3 (FJK-16 s) staining kit (eBiosciences, San Diego, CA). Intracellular staining for IL17A was performed using the intracellular fixation permeabilization kit and anti-mouse IL17 (TC11-18H10.1) specific antibodies from eBioscience™. Cells were also stained with antibodies to detect surface expression of CD45 (30-F11) and CD4 (clone GK1.5) to gate on the leukocyte population.

### T cell proliferation and cytokine assay

Mice were euthanized 10 days after immunization with PLP_91–110_ and draining lymph nodes were removed and challenged in vitro with PLP_91–110,_ as described previously [28]. The results are presented as stimulation indices, which are counts per minute (cpm) of test sample/cpm of the control). For cytokine analysis, supernatants were collected from culture 48 h after peptide stimulation, and the concentration of cytokines (IL-17F) were measured by sandwich ELISA using pairs of relevant anti-cytokine monoclonal antibodies (Pharmingen, San Diego, CA).

### Isolation of CD4^+^CD25^+^ Treg, CD4^+^CD25^−^ T effector, and dendritic cells

CD4^+^CD25^+^ T cells isolated from the spleens of DR3 and DR3.IL-17A^−/−^ mice using the EasySep™ Mouse CD4^+^CD25^+^ regulatory T Cell Isolation Kit II (STEMCELL Technologies, Vancouver, Canada) according to the manufacturer's protocols. CD4^+^CD25^−^ T effector cells were isolated from the Treg-depleted fraction of splenocytes using anti-Mouse CD4 Magnetic Particles – DM (BD Biosciences, CA, USA). DCs were isolated from total splenocytes using CD11c microbeads according to the manufacturer's protocols (BD Biosciences, CA, USA). The purity of specific cell populations was analyzed by flow cytometry; all populations used were > 90% pure.

### In vivo depletion of CD4^+^CD25^+^ Treg cells

DR3 and DR3.IL-17A^−/−^ mice received two intraperitoneal injections (200 µg/injection) of monoclonal anti-mouse CD25 (IL-2Ra) antibody (clone: PC-61.5.3; BioXcell) in 200 µl of PBS per dose on days −5 and −3 before induction of EAE [79]. Controls received two intraperitoneal injections of 200 µl of PBS. CD4^+^CD25^+^FoxP3^+^ Treg cell depletion was confirmed by flow cytometric analysis of PBMCs on the day EAE was induced. For IL-17A depletion, DR3 mice were injected intraperitoneally with 100 ug/mouse anti-IL17A (Clone 17F3; BioXcell), IgG1 (Clone MOPC-21; BioXcell) or PBS. IL-17A was blocked in C57BL/6 mice by administering 100 ug/mouse anti-IL-17A, IgG1 or PBS by intraperitoneally injection every three days following induction of EAE, starting at day 5 through day 32 (e.g., days −24, −21, −18, −15, −12, −9, −6 and −3 pre EAE induction and post EAE induction (at day 5, 8, 11, 14, and 17). IL-17F or GM-CSF cytokine inhibition was performed in vivo in DR3.IL-17A^−/−^ mice by intraperitoneally injection of 100 µg/mouse anti-IL-17F (clone; MM17F-8F5; BioXcell) or 100 µg/mouse of anti-GM-CSF (clone; MP1-22E9; BioXcell) post induction of EAE on days 5, 10, and 14. Control DR3.IL-17A^−/−^ mice received 100 µg/mouse IgG1 (Clone MOPC-21; BioXcell).

### Fecal Microbiota Transplant (FMT) and co-housing of DR3 and DR3.IL-17A^−/−^ mice

DR3.IL-17A^−/−^ mice were recipients of fecal transplants from DR3 mice through oral gavage, and conversely, DR3 mice were recipients of fecal transplants from DR3.IL-17A^−/−^ mice through oral gavage as per an established protocol [80]. Five days after oral gavage, both IL-17A suffcient and deficient DR3 strains were cohoused together. Fecal samples were collected before and after co-housing for shotgun metagenomic sequencing.

### Treg suppression assay

Spleens from 8–10-week-old DR3 and DR3.IL-17A^−/−^ mice were harvested for CD4^+^ T cells by BD™ IMag Particles as per manufacturer instruction. T cell-depleted splenocytes were used as antigen presenting cells after UV irradiation. CD4^+^CD25^+^ T cells were isolated from splenocytes of DR3 and DR3.IL-17A^−/−^ mice using EasySep™ Mouse CD4^+^CD25^+^ regulatory T cell isolation kit II (STEMCELL Technologies, Vancouver, Canada) according to the manufacturer's protocols. Treg suppression assays were performed as described previously [81].

### In vivo depletion of IL-17A in C57BL/6 mice

C57BL/6 mice were placed in three groups that received antibody injections prior to EAE induction (day −24, −21, −18, −15, −12, −9, −6, and −3): the first group of mice received intraperitoneal injections (200 µg/injection) of monoclonal anti-mouse IL-17A (clone 17F3; BioXCell) in 200 µl PBS per dose; the second group of mice received 200 µg/injection of isotype control (mouse IgG1) in 200 µl PBS per dose; and the third group of mice received 200 µl PBS to serve as vehicle control. Similarly, three groups of C57BL/6 mice were treated with either monoclonal anti-mouse IL-17A (200 µg/injection), isotype control mouse IgG1 (200 µg/injection), or PBS starting on day 5 post EAE induction, and continuing every third day until day 32. Mice were observed daily for disease symptoms and EAE was scored as per the scoring system described [61].

### Correlation analysis

*Prevotella*-related species enriched and transferred to DR3 from DR3.IL-17A^−/−^ mice and respective Treg population percentages quantified pre- and post-cohousing, *Prevotella*-related species and cumulative EAE scores in DR3 and DR3.IL-17A-/- mice pre- and post-cohousing and Treg population and cumulative EAE scores pre- and post-cohousing were correlated using spearman correlation.

## Supplementary Information

Supplementary Material 1.

## Data Availability

All data needed to evaluate the conclusions in the manuscript are present in the manuscript and/or the Supplementary Materials. Shotgun metagenomic sequencing and RNA-seq data had been uploaded under bio-project PRJNA808406. No custom codes were used for the data analysis.
