# Supplementary material for: IL-17A deficiency in HLA-DR3 transgenic mice enriches beneficial Prevotella species in gut to promote Tregs and reduce CNS autoimmunity
Source: Microbiome. 2026 Jun 29;14:176. doi: 10.1186/s40168-026-02394-w (PMC13326294; doi:10.1186/s40168-026-02394-w)
Supplement: Supplementary file 2 — Supplementary Material 1. [file 40168_2026_2394_MOESM1_ESM.pdf]

**Supplementary Figures**

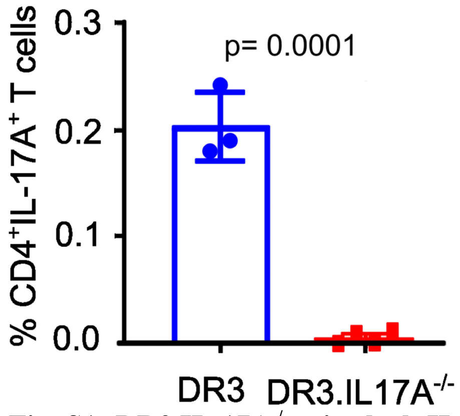


**Fig. S1: DR3.IL-17A$^{-/-}$ mice lack IL-17A-expressing CD4+ T cells in the spleen.** 8-10-week-old DR3 and DR3.IL-17A$^{-/-}$ transgenic mice were euthanized and splenocytes were harvested for analysis. Quantification of the frequency of CD4$^{+}$IL17A$^{+}$ T cells from mice in A. DR3: n=3; DR3.IL-17A$^{-/-}$, n = 4. *p*-value was determined using Mann-Whitney test between the experimental groups. Symbols represent individual mice, bars represent mean ± SEM.

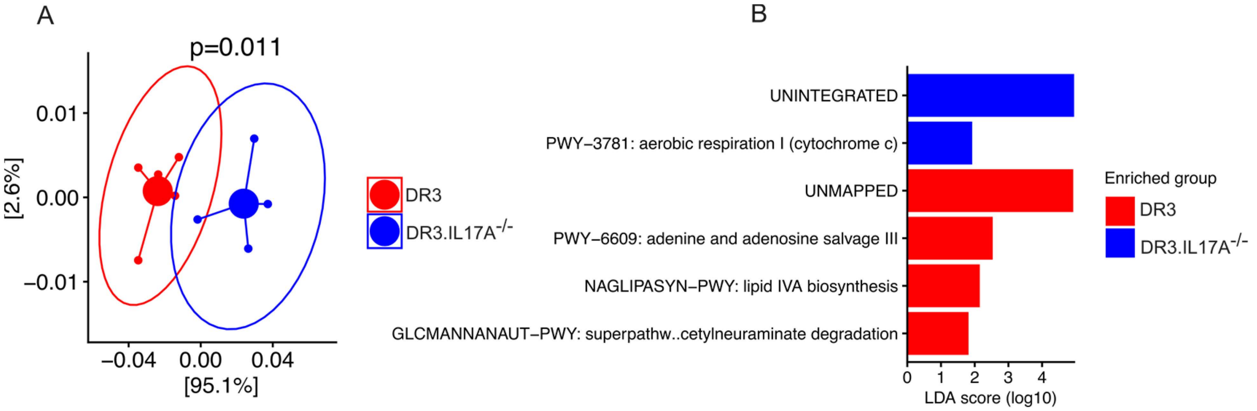


**Fig. S2: DR3 and DR3.IL-17A$^{-/-}$ transgenic mice harbor distinct functions in their gut microbiome.** Fecal samples were collected from 10-week-old DR3 (n=5) and DR3.IL-17A$^{-/-}$ (n=5) female mice. Gut microbiota composition was analyzed using shotgun metagenomic sequencing of fecal DNA. **(B)** Beta-diversity PCoA plot depicting clustering of functions between DR3 and DR3.IL-17A$^{-/-}$ gut microbiome using Bray-Curtis dissimilarity metric. *adonis2* test was performed for A. **(B)** Differential abundance of bacteria at species level using *lefse* from *microbiomeMarker* package at *kw_cutoff* =0.01, *wilcoxon_cutoff* =0.01 and *lda_cutoff* =1 in R.

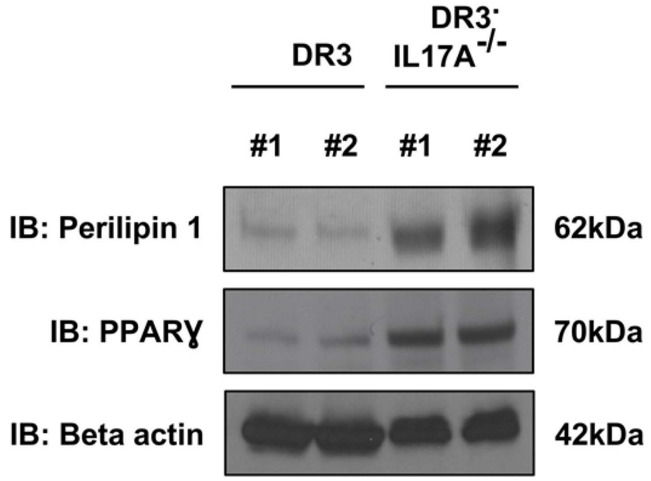


**Fig S3: Plin1 and PPARγ protein expression are increased in the colon of DR3.IL-17A$^{-/-}$ mice compared with DR3 controls.** Colon tissues from DR3 and DR3.IL-17A$^{-/-}$ mice were lysed in RIPA buffer containing protease inhibitors, and equal amounts of protein (50 µg) were separated by SDS-PAGE and transferred to PVDF membranes. Membranes were probed with Anti-Perilipin 1 (1:1000), Anti-PPARγ (1:1000), and Anti-β-actin (loading control). HRP-conjugated secondary antibodies were used (1:5000 for Perilipin 1 and PPARγ; 1:10,000 for β-actin), and signals were detected by chemiluminescence.

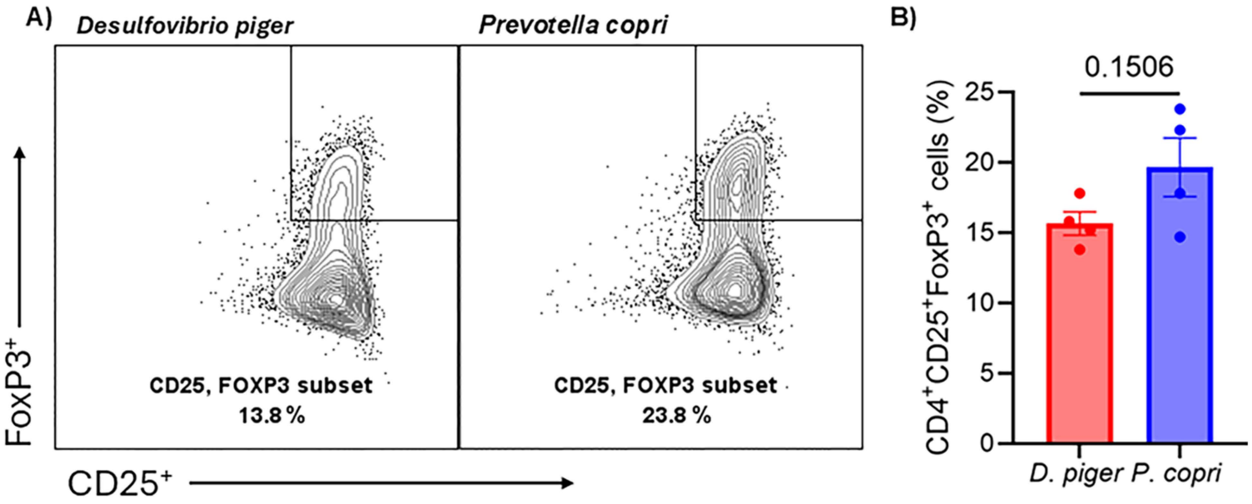


**Fig S4: Dendritic cells (DCs) primed with *P. copri* show an increasing trend in CD4$^+$CD25$^+$FoxP3$^+$ T cell differentiation compared with control bacteria primed DCs.** DCs were isolated from the spleens of DR3 mice using Miltenyi CD11c$^+$ selection beads and plated with plate-bound anti-CD3. Cells were stimulated overnight with *P. copri* or *D. piger* at a multiplicity of infection (MOI) of 10. On day 2, naïve CD4$^+$ T cells were isolated from DR3 spleens using a naïve CD4$^+$T cell isolation kit. The culture medium (complete RPMI with 10% FBS) was replaced, and purified naïve CD4$^+$ T cells were co-cultured with DCs at a 1:1 ratio in the presence of Treg differentiation cytokines IL-2 and TGF-β. On day 5, cells were restimulated with PMA and brefeldin A (BFA) for 3 hours, followed by surface and intracellular staining for flow cytometry. (**A**) Representative flow cytometry plots showing CD4$^+$CD25$^+$FoxP3$^+$ T cells in *P. copri* and *D. piger* primed DC co-cultures. (**B**) Quantification showing an increasing trend in CD4$^+$CD25$^+$FoxP3$^+$ T cell differentiation in *P. copri* primed DCs compared with *D. piger* primed controls (n = 4). *P* values were determined using Welch's *t* test.

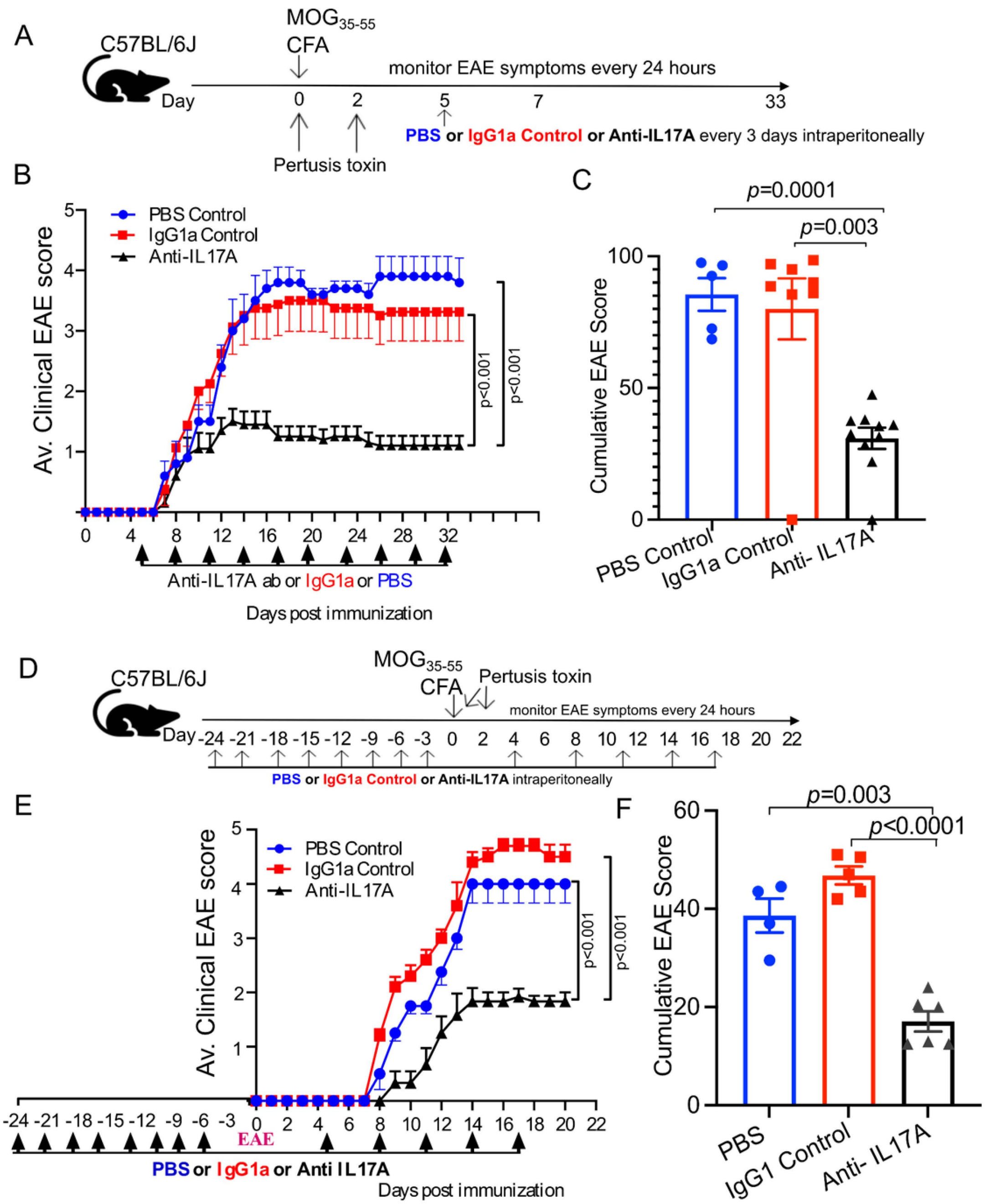


**Fig. S5: Anti-IL-17A treatment lowers the severity of $MOG_{35\text{-}55}$ induced EAE in C57BL/6J mice. (A-B)** 8-10-week-old B6 mice were immunized with $MOG_{35\text{-}55}$/CFA. Five days after immunization, mice received anti-mouse IL-17A monoclonal antibody, isotype control mouse IgG1, or PBS intraperitoneally every three days until the end of the experiment (day 32). (A) Average clinical EAE scores over time. Treatment days are indicated by arrowheads. (B) Average cumulative EAE scores. (**C-D**) 8-10-week-old B6 mice were treated every three days with anti-IL-17A, IgG1, or PBS pre- and post-immunization with $MOG_{35\text{-}55}$/CFA. **(C)** Average clinical EAE scores. Treatment days are indicated by arrowheads. **(D)** Average cumulative EAE scores. For plots in A, C, each bullet point represents the mean and bars represent the standard error of the mean from each experimental group. *p*-value was determined using two-way ANOVA following Tukey correction for EAE clinical scores (A, C) and unpaired t-test with Welch correction for cumulative EAE score (B, D). For graphs in B, D, each bar represents mean±SEM.

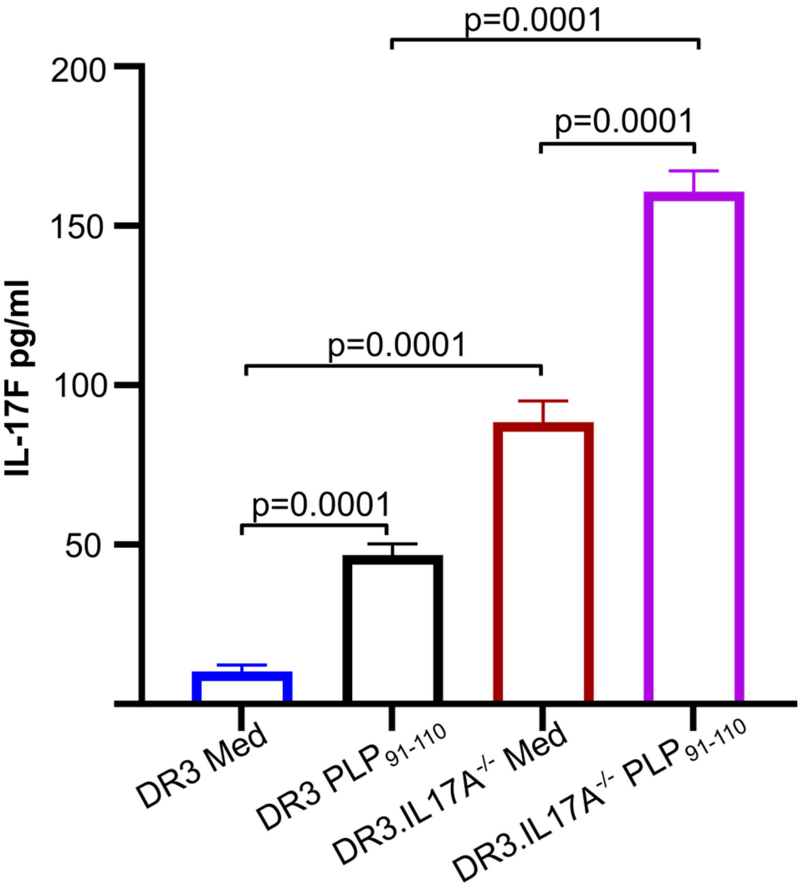


**Fig. S6: Levels of inflammatory cytokines IL-17F in DR3 and DR3.IL-17A$^{-/-}$ mice.** 8-10-week-old mice were immunized with $PLP_{91-110}$ /CFA to induce EAE. After 20 days, mice were euthanized, spleens were harvested, and splenic cells were cultured in the presence of $PLP_{91-110}$ antigen for five days following stimulation with Phorbol myristate acetate (PMA) and ionomycin in the presence of Brefeldin A (BFA). The supernatant was collected and levels of IL17F were measured using ELISA. Cytokine values from DR3 mice were compared to those of DR3.IL-17A$^{-/-}$ mice in either media (Med) or after stimulation with $PLP_{91-110}$. *p*-value was determined using unpaired t-test with Welch correction. Bars represent mean±S.E.M.

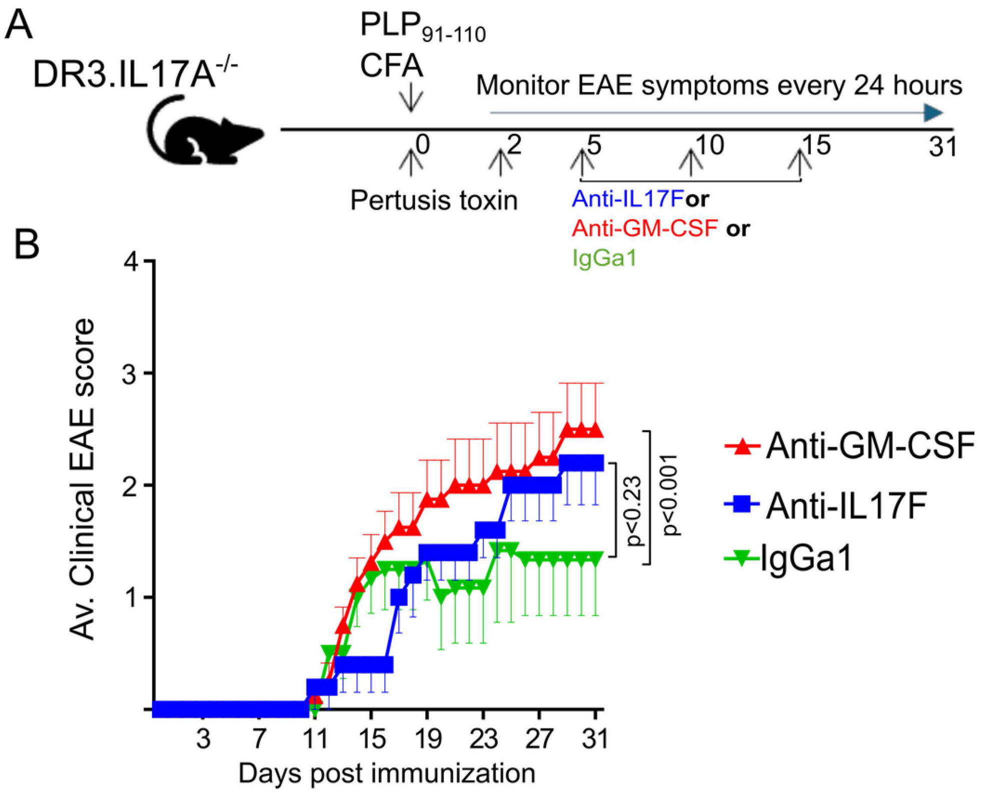


**Fig. S7: IL-17F and GM-CSF have no role in disease severity in** (**A**) Schematic diagram of the experimental workflow. EAE was induced in 8-10-week-old in DR3.IL-17A$^{-/-}$ transgenic mice. At days 5, 10, and 14 post-EAE induction, mice received intraperitoneal injections of either: 1) anti-GM-CSF monoclonal antibody, 2) anti-IL-17F monoclonal antibody, or 3) IgGa1 isotype control and clinical EAE scores were monitored for 31 days. (**B**) Average clinical EAE score of mice in each group over time. Bullet points represent the mean and bars represent the standard error of the mean of each experimental group from two independent experiments (n ≥3 mice per group). *p*-value was determined by a two-way ANOVA following Tukey correction for average clinical EAE score (B).

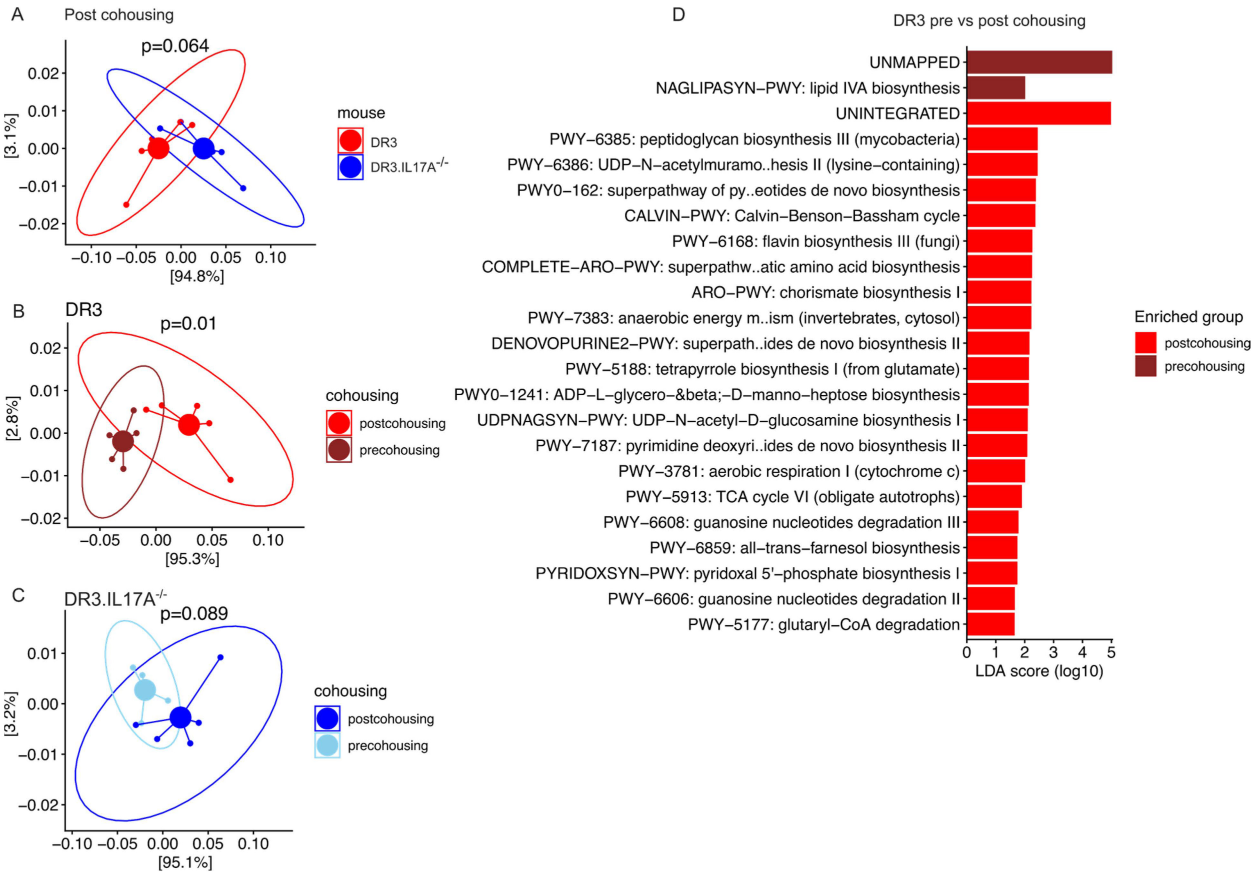


**Fig. S8: DR3.IL-17A$^{-/-}$ transfer functional pathways to DR3 transgenic mice.** Eight to ten-week-old DR3 (n = 5) and DR3.IL-17A$^{-/-}$ (n= 5) mice fecal samples were collected. Fecal from DR3 mice was transplanted to DR3.IL-17A$^{-/-}$ mice, and conversely, fecal materials from DR3.IL-17A$^{-/-}$ mice was transplanted into DR3 mice via oral gavage. Five days after oral gavage, DR3 mice were cohoused with DR3.IL-17A$^{-/-}$ mice. Fecal samples were collected before and after co-housing/fecal transplant for shotgun metagenomic sequencing. Humann3 was used with default parameters to profile the pathway abundances in the clean reads using MetaCyc databases. (**A**) Bray-Curtis dissimilarity metric-based PCoA plots showing microbiome functional composition between DR3 and DR3.IL-17A$^{-/-}$ post cohousing microbiome. **(B)** DR3 pre- and post-cohousing functions of the microbiome **(C)** DR3.IL-17A$^{-/-}$ pre- and post-cohousing functions of the microbiome. **(D)** Differential abundance of bacterial species using *lefse* from *microbiomeMarker* package at *kw_cutoff* =0.01, *wilcoxon_cutoff* =0.01 and *lda_cutoff* =1 in R for DR3 mice pre- and post-cohousing. *adonis2* test was performed for A, B and C.

**Supplemental Table:**

**Table S1:** Differentially expressed gene list in the colon of DR3.IL-17A$^{-/-}$ compared to DR3 transgenic mice calculated using *limma* package within *edgeR* in R. All 123 differentially expressed genes with *LogFC*>1.0 and *adj.p.val*<0.05 are shown.

| SN | Genes | logFC | AveExpr | t | P.Value | adj.P.Val |
|---|---|---|---|---|---|---|
| 1 | Eno1b | -12.154 | 1.443 | -19.576 | 0.000 | 0.000 |
| 2 | Hoxb13 | -11.448 | -1.890 | -19.897 | 0.000 | 0.000 |
| 3 | Hoxd13 | -9.589 | -1.902 | -10.871 | 0.000 | 0.000 |
| 4 | Casp14 | -8.793 | -1.658 | -13.128 | 0.000 | 0.000 |
| 5 | Glo1-ps | -8.601 | -0.491 | -9.588 | 0.000 | 0.000 |
| 6 | 2310079G19Rik | -8.148 | -2.842 | -8.433 | 0.000 | 0.000 |
| 7 | Rpl15-ps6 | -7.886 | 0.116 | -6.560 | 0.000 | 0.002 |
| 8 | Gm13237 | -7.823 | -0.170 | -8.445 | 0.000 | 0.000 |
| 9 | Ly6g | -7.548 | -2.237 | -11.113 | 0.000 | 0.000 |
| 10 | Tgm3 | -6.671 | 3.560 | -31.635 | 0.000 | 0.000 |
| 11 | Vtcn1 | -6.393 | -3.095 | -8.575 | 0.000 | 0.000 |
| 12 | Mettl7a2 | -6.307 | -3.303 | -8.664 | 0.000 | 0.000 |
| 13 | Igkv8-18 | -6.293 | -0.666 | -8.186 | 0.000 | 0.000 |
| 14 | Fxyd4 | -6.059 | -1.910 | -4.809 | 0.000 | 0.025 |
| 15 | 5033404E19Rik | -5.855 | -2.774 | -5.566 | 0.000 | 0.008 |
| 16 | Gm7334 | -5.659 | 1.342 | -5.672 | 0.000 | 0.007 |
| 17 | Sval1 | -5.631 | 3.704 | -19.493 | 0.000 | 0.000 |
| 18 | Evx2 | -5.587 | -2.795 | -6.518 | 0.000 | 0.002 |
| 19 | Gm8540 | -5.532 | -3.496 | -7.667 | 0.000 | 0.000 |
| 20 | Atp12a | -5.477 | 0.033 | -18.752 | 0.000 | 0.000 |
| 21 | Chit1 | -4.989 | -3.144 | -5.998 | 0.000 | 0.004 |
| 22 | Trpv6 | -4.912 | -0.557 | -8.873 | 0.000 | 0.000 |
| 23 | Igkv2-112 | -4.725 | 1.275 | -9.585 | 0.000 | 0.000 |
| 24 | Fut9 | -4.701 | -1.951 | -6.631 | 0.000 | 0.002 |
| 25 | Ighv7-1 | -4.595 | 0.648 | -6.919 | 0.000 | 0.001 |
| 26 | Ninj2 | -4.593 | -1.906 | -5.754 | 0.000 | 0.006 |
| 27 | St8sia5 | -4.551 | -1.819 | -5.220 | 0.000 | 0.013 |
| 28 | Ighv1-61 | -4.532 | 1.365 | -7.272 | 0.000 | 0.001 |
| 29 | Tmprss13 | -4.269 | -0.183 | -8.813 | 0.000 | 0.000 |
| 30 | Gjb5 | -4.261 | -1.541 | -4.778 | 0.000 | 0.025 |
| 31 | Hoxd12 | -4.259 | -3.247 | -4.345 | 0.000 | 0.049 |
| 32 | Vsig1 | -4.255 | -1.212 | -6.516 | 0.000 | 0.002 |
| 33 | Ighv10-3 | -4.253 | 3.640 | -11.518 | 0.000 | 0.000 |
| 34 | B3gnt7 | -4.195 | 5.973 | -34.648 | 0.000 | 0.000 |

| | | | | | | |
|---|---|---|---|---|---|---|
| 35 | Rpl15-ps2 | -3.829 | -0.635 | -4.963 | 0.000 | 0.019 |
| 36 | Itih2 | -3.817 | -1.521 | -4.471 | 0.000 | 0.041 |
| 37 | Mettl7a3 | -3.571 | -1.774 | -4.445 | 0.000 | 0.042 |
| 38 | Pla2g4f | -3.458 | 0.531 | -5.797 | 0.000 | 0.006 |
| 39 | Trpv3 | -3.403 | 3.252 | -12.943 | 0.000 | 0.000 |
| 40 | Thbs4 | -3.334 | 3.057 | -21.508 | 0.000 | 0.000 |
| 41 | Hoxa13 | -3.280 | -1.700 | -8.765 | 0.000 | 0.000 |
| 42 | Itln1 | -3.187 | 6.879 | -10.731 | 0.000 | 0.000 |
| 43 | Sycn | -3.151 | 2.937 | -24.593 | 0.000 | 0.000 |
| 44 | Nxpe4 | -3.057 | 3.610 | -15.040 | 0.000 | 0.000 |
| 45 | Insl5 | -2.941 | -1.314 | -6.488 | 0.000 | 0.002 |
| 46 | Ighv2-5 | -2.897 | 2.075 | -4.897 | 0.000 | 0.021 |
| 47 | Adgrf1 | -2.818 | 0.607 | -9.433 | 0.000 | 0.000 |
| 48 | Igkv3-4 | -2.711 | 3.280 | -8.454 | 0.000 | 0.000 |
| 49 | Igkv4-57 | -2.655 | 4.588 | -12.494 | 0.000 | 0.000 |
| 50 | Slc28a3 | -2.572 | 2.546 | -4.625 | 0.000 | 0.032 |
| 51 | Igkv4-68 | -2.565 | 4.316 | -8.356 | 0.000 | 0.000 |
| 52 | Pla2g2f | -2.494 | 3.879 | -7.097 | 0.000 | 0.001 |
| 53 | Ighv3-3 | -2.458 | 1.456 | -8.997 | 0.000 | 0.000 |
| 54 | Cyp2d12 | -2.407 | 1.294 | -9.324 | 0.000 | 0.000 |
| 55 | Best2 | -2.363 | 0.866 | -10.732 | 0.000 | 0.000 |
| 56 | Gm47283 | -2.290 | 5.085 | -10.117 | 0.000 | 0.000 |
| 57 | Adh1 | -2.274 | 5.559 | -21.681 | 0.000 | 0.000 |
| 58 | Ighv1-39 | -2.271 | 3.886 | -4.873 | 0.000 | 0.022 |
| 59 | Cyp2c68 | -2.248 | 4.529 | -8.006 | 0.000 | 0.000 |
| 60 | B3gnt5 | -2.203 | 3.517 | -6.569 | 0.000 | 0.002 |
| 61 | Ighv5-4 | -2.151 | 2.640 | -5.001 | 0.000 | 0.018 |
| 62 | Ggh | -2.100 | 5.137 | -16.144 | 0.000 | 0.000 |
| 63 | Cyp2d9 | -2.078 | 0.066 | -4.490 | 0.000 | 0.040 |
| 64 | Zfp791 | -2.041 | 2.587 | -4.591 | 0.000 | 0.034 |
| 65 | Slc15a2 | -2.026 | 2.158 | -4.924 | 0.000 | 0.020 |
| 66 | Nox1 | -2.008 | 3.528 | -7.361 | 0.000 | 0.001 |
| 67 | Nt5e | -1.973 | 5.284 | -10.629 | 0.000 | 0.000 |
| 68 | Duoxa2 | -1.952 | 6.303 | -10.058 | 0.000 | 0.000 |
| 69 | Ang4 | -1.951 | 7.834 | -9.720 | 0.000 | 0.000 |
| 70 | Ighv2-9-1 | -1.949 | 3.374 | -4.962 | 0.000 | 0.019 |
| 71 | Slc15a1 | -1.948 | 6.682 | -8.009 | 0.000 | 0.000 |
| 72 | Igkv3-5 | -1.942 | 3.424 | -5.541 | 0.000 | 0.008 |
| 73 | Cldn8 | -1.909 | 4.312 | -9.203 | 0.000 | 0.000 |

| | | | | | | |
|---|---|---|---|---|---|---|
| 74 | Eno3 | -1.762 | 3.750 | -6.374 | 0.000 | 0.002 |
| 75 | Clic6 | -1.762 | 3.833 | -5.762 | 0.000 | 0.006 |
| 76 | Cd177 | -1.749 | 6.807 | -16.983 | 0.000 | 0.000 |
| 77 | Adamts15 | -1.691 | 4.728 | -5.747 | 0.000 | 0.006 |
| 78 | Ccl28 | -1.628 | 7.068 | -12.416 | 0.000 | 0.000 |
| 79 | Igkv9-120 | -1.622 | 4.390 | -4.733 | 0.000 | 0.027 |
| 80 | Hoxa11 | -1.621 | 0.018 | -5.452 | 0.000 | 0.009 |
| 81 | Ceacam10 | -1.593 | 5.986 | -8.932 | 0.000 | 0.000 |
| 82 | Aqp4 | -1.573 | 6.556 | -14.118 | 0.000 | 0.000 |
| 83 | Saa1 | -1.550 | 7.353 | -7.683 | 0.000 | 0.000 |
| 84 | Ano1 | -1.550 | 3.699 | -6.163 | 0.000 | 0.003 |
| 85 | Tcf7l1 | -1.529 | 2.331 | -4.795 | 0.000 | 0.025 |
| 86 | Cela1 | -1.499 | 4.613 | -7.343 | 0.000 | 0.001 |
| 87 | Gm15401 | -1.437 | 3.493 | -4.550 | 0.000 | 0.036 |
| 88 | Prdx6 | -1.317 | 8.165 | -8.030 | 0.000 | 0.000 |
| 89 | Retnlb | -1.259 | 5.150 | -4.445 | 0.000 | 0.042 |
| 90 | Scd2 | -1.133 | 9.600 | -5.131 | 0.000 | 0.015 |
| 91 | Plin4 | 1.287 | 6.266 | 4.615 | 0.000 | 0.033 |
| 92 | Cyp4b1 | 1.379 | 4.923 | 4.760 | 0.000 | 0.026 |
| 93 | Slc13a1 | 1.393 | 7.159 | 5.688 | 0.000 | 0.006 |
| 94 | Hbb-bs | 1.412 | 5.567 | 5.082 | 0.000 | 0.016 |
| 95 | Naaladl1 | 1.451 | 8.878 | 5.901 | 0.000 | 0.005 |
| 96 | Oas2 | 1.473 | 3.640 | 4.420 | 0.000 | 0.044 |
| 97 | Tef | 1.533 | 4.008 | 5.337 | 0.000 | 0.011 |
| 98 | Oasl2 | 1.568 | 5.620 | 8.209 | 0.000 | 0.000 |
| 99 | Fabp4 | 1.636 | 8.267 | 8.666 | 0.000 | 0.000 |
| 100 | Ifit1 | 1.636 | 6.835 | 13.024 | 0.000 | 0.000 |
| 101 | Rpl3 | 1.638 | 7.213 | 9.483 | 0.000 | 0.000 |
| 102 | Thrsp | 1.641 | 5.122 | 4.949 | 0.000 | 0.020 |
| 103 | Ddx60 | 1.649 | 4.067 | 6.229 | 0.000 | 0.003 |
| 104 | Ifit3 | 1.670 | 4.893 | 6.902 | 0.000 | 0.001 |
| 105 | Retn | 1.716 | 4.432 | 5.022 | 0.000 | 0.018 |
| 106 | Sis | 1.727 | 7.589 | 5.489 | 0.000 | 0.009 |
| 107 | Igkv12-98 | 1.757 | 2.715 | 4.414 | 0.000 | 0.044 |
| 108 | Oas3 | 1.774 | 4.742 | 8.831 | 0.000 | 0.000 |
| 109 | Dbp | 1.855 | 4.802 | 9.977 | 0.000 | 0.000 |
| 110 | Rpl15 | 1.893 | 7.014 | 8.315 | 0.000 | 0.000 |
| 111 | Igkv14-100 | 1.910 | 3.744 | 5.128 | 0.000 | 0.015 |
| 112 | Car3 | 2.300 | 7.529 | 24.744 | 0.000 | 0.000 |

| | | | | | | |
|---|---|---|---|---|---|---|
| 113 | Cfd | 2.334 | 7.125 | 24.230 | 0.000 | 0.000 |
| 114 | Adipoq | 2.474 | 4.756 | 11.111 | 0.000 | 0.000 |
| 115 | Plin1 | 2.574 | 4.541 | 7.430 | 0.000 | 0.001 |
| 116 | Lgals2 | 2.688 | 5.834 | 6.063 | 0.000 | 0.004 |
| 117 | Tcaim | 2.984 | 2.326 | 6.378 | 0.000 | 0.002 |
| 118 | Trgc2 | 2.984 | 1.211 | 4.446 | 0.000 | 0.042 |
| 119 | Cyp2e1 | 3.009 | 5.145 | 15.014 | 0.000 | 0.000 |
| 120 | Lyrm7 | 3.114 | 0.716 | 4.632 | 0.000 | 0.032 |
| 121 | Gm16011 | 4.232 | -0.321 | 4.370 | 0.000 | 0.047 |
| 122 | Igkv14-126 | 5.013 | 0.842 | 7.976 | 0.000 | 0.000 |
| 123 | Cylc2 | 8.644 | -1.325 | 9.999 | 0.000 | 0.000 |

**Table S2. Effect of *Prevotella copri* (*P. copri*) DSM 18205 supplementation on PPAR pathways genes, FoxP3, and IL10 gene expression in the colon of DR3 mice**

| | DR3 | | | | | | DR3IL17A | | | | Unpaired T-test with Welch correction |
|---|---|---|---|---|---|---|---|---|---|---|---|
| Plin1 | 0.001 | 0.004 | 0.007 | 0.002 | | 0.011 | 0.016 | 0.011 | 0.007 | | p=0.0123 |
| Plin4 | 0.067 | 0.061 | 0.071 | 0.053 | | 0.103 | 0.103 | 0.093 | 0.111 | | p=0.0003 |
| Adipoq | 0.407 | 1.879 | 4.558 | 0.287 | | 4.552 | 7.031 | 7.363 | 0.347 | | p=0.17 |
| FABP4 | 0.638 | 1.919 | 2.078 | 0.411 | | 3.040 | 3.758 | 3.026 | 1.772 | | p=0.033 |
| | | | | | | | | | | | |
| | Media | | | | | | P. copri | | | | |
| IL10 | 0.200 | 3.900 | 0.600 | 0.800 | 2.700 | 5.000 | | 0.500 | 19.100 | 23.500 | p=0.15 |
| FoxP3 | 3.800 | 0.100 | 3.700 | 0.900 | 0.800 | 0.200 | 0.100 | 9.100 | 6.100 | 11.700 | p=0.2 |
| FABP4 | 2.127 | 0.923 | 0.992 | 1.340 | 0.384 | 2.105 | 2.456 | 1.093 | 1.921 | 2.762 | p=0.053 |
| Plin1 | 1.448 | 1.008 | 0.685 | 3.279 | 0.305 | 2.790 | 6.372 | 1.096 | 1.494 | 2.237 | p=0.22 |
| Plin4 | 1.050 | 0.700 | 2.548 | 0.441 | 1.211 | 0.738 | 1.734 | 1.239 | 0.691 | 0.949 | p=0.78 |
| Adipoq | 3.238 | 1.341 | 1.204 | 1.526 | 0.125 | 1.849 | 3.462 | 1.862 | 1.881 | 4.438 | p=0.13 |
